# Estimation of the breadth of CD4bs targeting HIV antibodies by molecular modeling and machine learning

**DOI:** 10.1371/journal.pcbi.1006954

**Published:** 2019-04-10

**Authors:** Simone Conti, Martin Karplus

**Affiliations:** 1 Department of Chemistry and Chemical Biology, Harvard University, Cambridge, Massachusetts, United States of America; 2 Laboratoire de Chimie Biophysique, ISIS, Université de Strasbourg, Strasbourg, France; US Army Medical Research and Materiel Command, UNITED STATES

## Abstract

HIV is a highly mutable virus for which all attempts to develop a vaccine have been unsuccessful. Nevertheless, few long-infected patients develop antibodies, called broadly neutralizing antibodies (bnAbs), that have a high breadth and can neutralize multiple variants of the virus. This suggests that a universal HIV vaccine should be possible. A measure of the efficacy of a HIV vaccine is the neutralization breadth of the antibodies it generates. The breadth is defined as the fraction of viruses in the Seaman panel that are neutralized by the antibody. Experimentally the neutralization ability is measured as the half maximal inhibitory concentration of the antibody (IC_50_). To avoid such time-consuming experimental measurements, we developed a computational approach to estimate the IC_50_ and use it to determine the antibody breadth. Given that no direct method exists for calculating IC_50_ values, we resort to a combination of atomistic modeling and machine learning. For each antibody/virus complex, an all-atoms model is built using the amino acid sequence and a known structure of a related complex. Then a series of descriptors are derived from the atomistic models, and these are used to train a Multi-Layer Perceptron (an Artificial Neural Network) to predict the value of the IC_50_ (by regression), or if the antibody binds or not to the virus (by classification). The neural networks are trained by use of experimental IC_50_ values collected in the CATNAP database. The computed breadths obtained by regression and classification are reported and the importance of having some related information in the data set for obtaining accurate predictions is analyzed. This approach is expected to prove useful for the design of HIV bnAbs, where the computation of the potency must be accompanied by a computation of the breadth, and for evaluating the efficiency of potential vaccination schemes developed through modeling and simulation.

## Author summary

Although we are now almost 40 years into the AIDS epidemics, no approved vaccine for HIV exists. This is due to the high mutability of HIV, which allows it to escape the immune system control. Nevertheless, a few long-infected patients have been able to develop antibodies, called broadly neutralizing antibodies (bnAbs), that have a high neutralization breadth and are effective against a variety of viral strains. The knowledge that bnAbs can develop over time suggests that a HIV vaccine can be found that short-circuits the production of bnAbs. In this study we present a computational approach to estimate the breadth of HIV antibodies. Experimentally, the breadth is the fraction of viruses in a panel that are neutralized by the antibody, where the ability to neutralize is quantified as the half maximal inhibitory concentration of the antibody (IC_50_). A method to estimate the IC_50_ by computer modeling and machine learning is described and used for estimating the antibody breadth. This approach is likely to prove useful in the design of new antibodies effective against HIV and for testing the efficiency of theoretically-designed vaccination protocols.

## Introduction

Vaccination is a medical procedure which has played an essential role in protecting mankind against viral and bacterial infections since the time of Edward Jenner, who developed a vaccine for smallpox over 200 years ago. Although for some diseases caused by viruses, such as measles, a small number of vaccinations provide almost permanent immunity, for other such as influenza, an annual revaccination, which provides only limited protection, is required. Since the measles virus, like the flu virus, undergoes error-prone replication that introduces mutations, it is not clear why the measles vaccination works as well as it does. Recent research [1] suggests that the measles virus remains antigenically monotypic because mutations are almost always lethal, though the reason for that is not known.

For HIV, which is the focus of this paper, no approved vaccine exists, although we are now almost forty years into the HIV/AIDS epidemic. As is the case for influenza virus, HIV evolves rapidly so that there exist many different viral strains. Some of them can evade the immune response to a vaccination directed against only a small number of strains. Thanks to the development of antiretroviral therapies [2], people who are infected by HIV can live essentially normal lives, without succumbing to AIDS. Several years after infection, a small fraction of the HIV infected individuals develop antibodies that are referred to as broadly neutralizing antibodies (bnAbs), i.e. antibodies that are effective against many strains of the virus [3]; an example of a detailed structural study of the germline and mature bnAbs from a single patient is given in Fera *et al*. [4]. The fact that the immune system can develop bnAbs over time has led to renewed interest in the possibility of developing a vaccine that would be effective against HIV [5,6].

The “neutralization breadth” of an antibody is a measure of its ability to recognize and neutralize different variants of the virus. An antibody with a high breadth can recognize effectively many different variants, while a low breadth antibody is more specific. Experimentally, the neutralization breadth of an antibody is evaluated by testing its ability to inhibit a panel of antigens from different clades of the target virus [7]. In what follows we simply write “breadth” when referring to “neutralization breadth”. For HIV, the Seaman virus panel contains 109 representatives of HIV clades A, B, C and circulating recombinant forms [7]. To evaluate the breadth of an antibody, the half maximal inhibitory concentration of the antibody (IC_50_) is measured for each virus in the panel. The breadth is defined as the fraction of viruses for which the IC_50_ is less than a given cutoff, usually set to 1 μg/ml.

The present paper describes a computational method to estimate the breadth of new HIV antibodies using only the sequence of their heavy and light chains, and the assumption that they form antibody/antigen complexes that are similar to a known crystal structure of an antibody/antigen complex that may not have high sequence homology. Of the known HIV antibodies, we select those that target the CD4 binding site (CD4bs) of the HIV Envelope glycoprotein, a binding site used by diverse bnAbs [5]. Because the CD4bs is highly conserved, an HIV vaccine designed to elicit bnAbs that bind to this site would have a high therapeutic potential: bnAbs would likely recognize the conserved core, as well as the variable regions in the neighborhood that are required for the broad-based character to develop [8].

**Fig 1:**
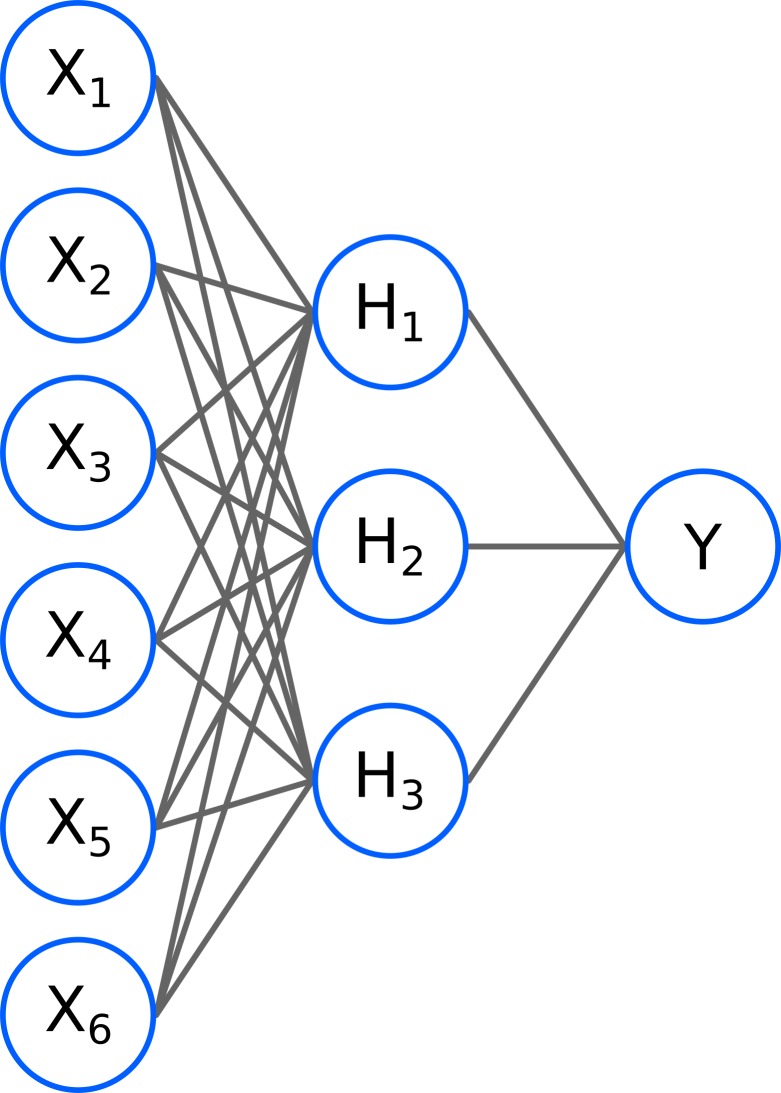
Generic structure of an Artificial Neural Network. It is composed of an input layer (the X_i_ nodes) that contains the descriptors developed for the system being studied, a hidden layer (H_i_ nodes) that combines non-linearly the descriptors in the input layer (through a logistic activation function), and an output layer (Y), that again combines non-linearly the nodes in the hidden layer and produces the output result.

However, a major obstacle to a successful computational design of bnAbs is a lack of accurate methods for computing the antibody breadth. Here, we use machine learning [9] coupled with descriptors obtained from all-atoms models of the antibody/antigen complex to predict the IC_50_ values for the viruses in the Seaman panel from which the antibody breadth can be estimated. To predict the IC_50_ we use a supervised artificial neural network [10,11], a multilayer perceptron (MLP); see Fig 1. The neural network is trained by backpropagation, where the errors in the outputs are propagated backwards into the artificial network structure to optimize the internal parameters of the model. An essential element for the successful application of artificial neural networks is the availability of a large data set for training the networks [11]. For HIV, experimental IC_50_ values have been collected in the CATNAP database of neutralizing antibodies [12], which contains more than 40000 IC_50_ values; of these, 6179 satisfy the criteria required for our machine learning approach.

In the body of the paper, we describe training of the MLP to predict either the actual value of the IC_50_ (by regression), or whether or not the antibody recognizes the virus (by classification). The outputs of the two artificial neural networks are then used to estimate the breadth of known antibodies. In a Concluding Discussion, we consider the limitation and extension of the methodology described here, as well as its utility for vaccine design.

## Results

### Neural network regression model for the IC_50_

The first step toward the estimation of the breadth of a given antibody is to evaluate the IC_50_ for the binding of the antibody with all the antigens in the Seaman virus panel [7]. Here, the IC_50_ values are estimated using a Neural Network regression model (an MLP regressor), trained over experimental IC_50_ values. Experimental values are obtained from the CATNAP database, which contains a total of about 40000 IC_50_ values [12]. The values in the database were filtered to make sure that the amino acid sequences of both the antibody and antigen are available, that at least one crystallographic structure of the antibody bound to a related HIV antigen is known, and that the antibody binds to the CD4 binding site (CD4bs). Upon filtering the database for these attributes, a total of 6179 IC_50_ values were obtained. Of these, 3864 are reported as exact values, while the remaining 2315 are reported as “greater than” a given cutoff (e.g. “>50 μg/ml”); see Table 1. We refer to these as “approximated” values. To train the MLP regression model, only the 3864 exact values are used; the approximate ones were discarded.

**Table 1:** Antibodies studied in this work. The second and third columns report the number of experimental IC_50_ values available as exact or approximate values. The fourth column reports the experimental breadth calculated based on the Seaman antigen panel. The number in parenthesis is the number of antigens used to calculate the breadth (the Seaman panel contains 109 antigens, but the experimental IC_50_ value is not available for all of them). The fifth column indicates the PDB ID used as the template for modeling the antibody/antigen complex. If only one PDB is indicated, that is used for the modeling “as is”. If two are present, the first is used to model the antibody, the second for the antigen (after best-fit RMSD superimposition to the antigen in the first PDB). The last three columns report the breadth calculated using the IC_50_ regressor with and without approximated values and using the IC_50_ classifier (see text).

Antibody	Exact	Exact and approximate	Breadth (N. Ag)	PDB	Calculated Breadth		
					IC_50_ regressor w/o approx.	IC_50_ regressor w/ approx.	IC_50_ classifier
12A21	17	26	0.46 (13)	4JPW/5FYJ	0.44±0.14	0.10±0.09	0.38±0.13
1B2530	74	171	0.11 (76)	4YFL/5FYJ	0.05±0.05	0.00±0.00	0.03±0.04
3BNC117	351	444	0.78 (87)	4LSV/5FYJ	0.81±0.05	0.54±0.08	0.80±0.05
8ANC131	136	178	0.32 (76)	4RWY/5FYJ	0.13±0.06	0.03±0.03	0.22±0.09
8ANC134	86	169	0.31 (75)	4RX4/5FYJ	0.15±0.09	0.03±0.03	0.25±0.10
b12	242	690	0.18 (109)	2NY7/5D9Q	0.05±0.03	0.00±0.00	0.03±0.02
b13	18	176	0.03 (76)	2NY7/5D9Q	0.09±0.08	0.00±0.00	0.02±0.02
CAP257-RH1	1	191	0.01 (77)	5T33/5FYJ	0.61±0.30	0.00±0.00	0.00±0.00
CH103	162	191	0.39 (79)	4JAN/5FYJ	0.16±0.09	0.08±0.04	0.39±0.13
CH235	37	196	0.00 (77)	5F9W/5FYJ	0.02±0.02	0.00±0.00	0.00±0.01
CH235.12	174	194	0.64 (77)	5F96/5FYJ	0.20±0.09	0.06±0.05	0.48±0.14
HJ16	87	252	0.16 (97)	4YE4/5FYJ	0.24±0.08	0.00±0.00	0.06±0.06
IOMA	59	117	0.19 (53)	5T3X	0.17±0.10	0.01±0.01	0.10±0.06
N6	341	350	0.97 (76)	5TE6/5FYJ	0.96±0.02	0.89±0.04	0.96±0.03
NIH45-46	222	265	0.77 (86)	3U7Y/5FYJ	0.83±0.06	0.43±0.13	0.81±0.06
VRC01	501	609	0.70 (107)	5FYJ	0.48±0.14	0.21±0.10	0.79±0.08
VRC03	178	318	0.45 (107)	3SE8/5FYJ	0.36±0.09	0.02±0.02	0.28±0.06
VRC06	65	176	0.16 (76)	4JB9/5FYJ	0.27±0.09	0.01±0.01	0.16±0.05
VRC06b	80	174	0.31 (74)	4XNZ/5FYJ	0.35±0.18	0.01±0.02	0.22±0.15
VRC07	353	389	0.84 (87)	4OLU/5FYJ	0.85±0.05	0.69±0.08	0.91±0.04
VRC23	109	175	0.16 (76)	4J6R/5FYJ	0.04±0.02	0.00±0.01	0.05±0.05
VRC-CH31	213	265	0.67 (87)	4LSP/5FYJ	0.58±0.09	0.30±0.09	0.66±0.06
VRC-PG04	220	285	0.64 (86)	3J5M	0.65±0.09	0.31±0.09	0.65±0.06
VRC-PG20	138	178	0.58 (76)	4LSU/5FYJ	0.71±0.06	0.35±0.15	0.75±0.10
Total	3864	6179					

As inputs for the MLP regressor, we use the values of descriptors of the antibody/antigen bound system obtained from an atomistic model of the complex. A total of 24 descriptors were tested; they fall into four classes: atomic descriptors, protein/protein scoring functions, protein stability scoring functions, and entropy models (see Methods). The procedure for building the model for one antibody/antigen complex and evaluating all the descriptors requires about 13 minutes (see Methods); for evaluating the values of the subset of 19 chosen descriptors only about 20 seconds are required, once the model has been constructed (see Methods).

For training the MLP regressor the available experimental IC_50_ values are randomly divided in half to obtain a training set and a test set. The training is then performed, and the obtained neural network used to predict the IC_50_. The training is repeated 30 times using each time a randomly generated training and validation set, and the results are averaged over the 30 neural network results to estimate the error in the predictions.

**Fig 2:**
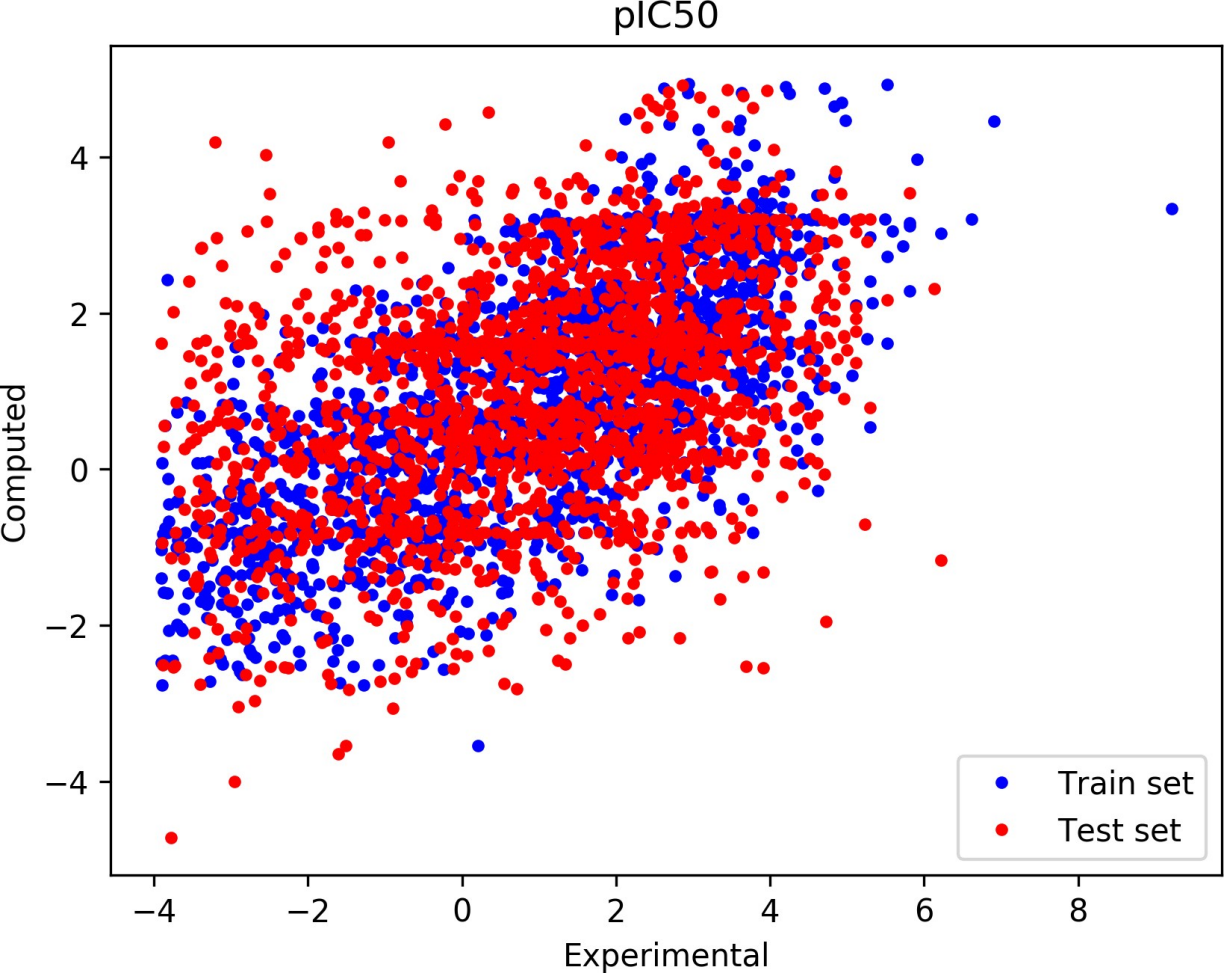
Correlation between the computed and experimental pIC_50_ (= −logIC_50_). Blue dots represent the set of data used to fit the MLP regressor, the red set represents the validation (or test) set of values. The Pearson correlation coefficient for training set is 0.686 (Spearman coefficient is 0.682), and it decreases to 0.410 (Spearman 0.408) for the validation set.

**Fig 3:**
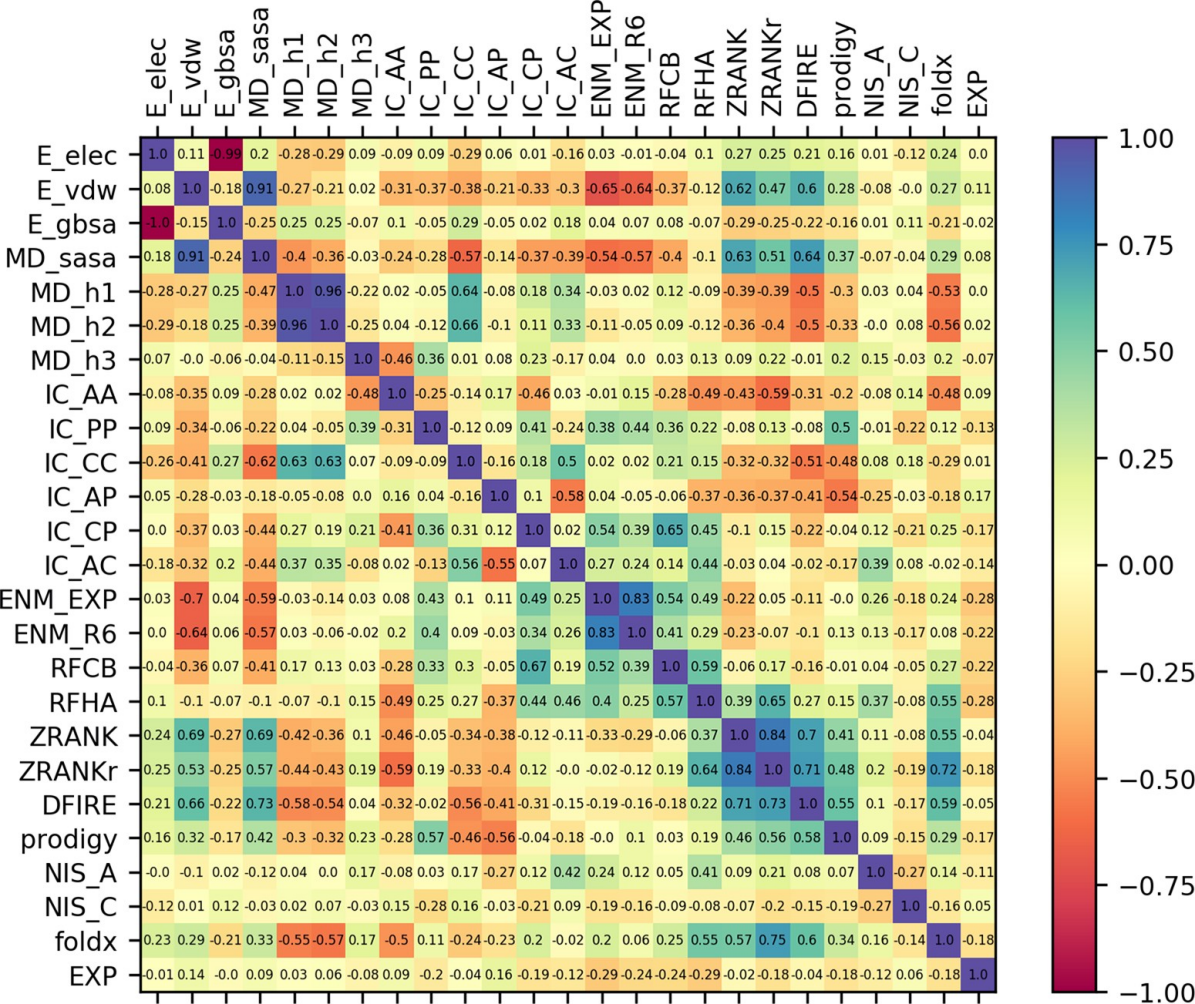
Cross correlation between all descriptors and the experimental IC_50_ values. The upper triangular reports the Pearson coefficients, the lower triangular the Spearman coefficients. The last column/row report the correlation with the experimental values.

The correlation between the experimental and computed IC_50_ is shown in Fig 2 for both the training and the test sets. The Pearson correlation coefficient for the training set is 0.686 (Spearman coefficient is 0.682). For the test set, the correlation coefficients decrease to 0.410 (Pearson) and 0.408 (Spearman). This is a significant improvement over the use of individual descriptors, which maximally reach a Pearson correlation coefficient of 0.28; see Fig 3 (last column).

**Fig 4:**
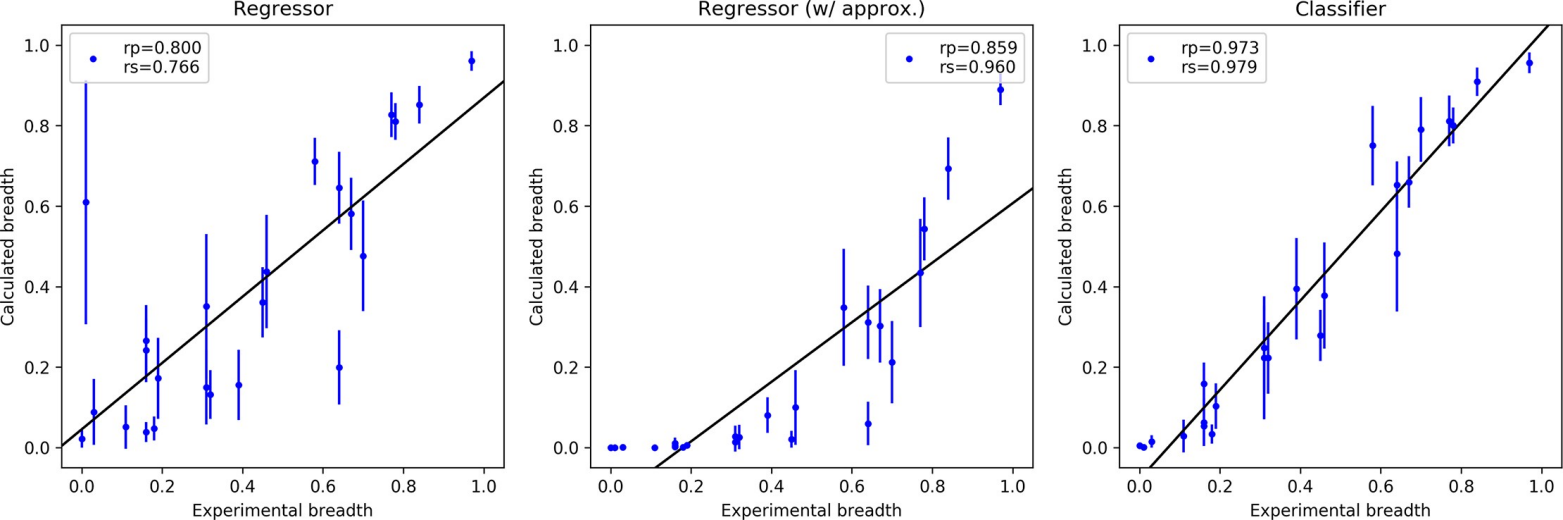
Comparison between experiments and predicted neutralization breadth for 24 antibodies evaluated using the IC_50_ values obtained via the MLP regressor without approximate values (left), the MLP regressor with approximate values (center), and the MLP classifier (right).

With these IC_50_ values, the breadth of known antibodies can be computed and compared with the experimental values. In the CATNAP database, 24 antibodies have a known sequence and bind to the CD4bs with a known crystallographic structure; see Table 1. The breadth is calculated for all of these using the computed IC_50_ values for the viruses in the Seaman panel. Specifically, for each virus in the Seaman panel, the IC_50_ is estimated using the MLP regressor, and the fraction of viruses for which the IC_50_ is less than 1 μg/ml is counted. Fig 4 (left) compares the experimental and computed breadths and their estimated errors (see Methods). For most antibodies the predicted breadth is in reasonable agreement with the experiments, although there is a tendency to overestimate it. The Pearson correlation coefficient is 0.800 (Spearman is 0.766) indicating a meaningful correlation. The breadth of the antibody CAP257-RH1 is significantly overestimated (experimental is 0.013, computed is 0.61±0.30), but this antibody is the one for which only a single exact IC_50_ value is available, and the computed uncertainty is highest.

Given the limited accuracy for the prediction of the IC_50_ values (Fig 2), this result is particularly encouraging. It indicates a low sensitivity of the breadth to the actual IC_50_ values. This is rooted in the definition of the breadth, which does not require the exact value of the IC_50_, but only whether the antibody does or does not bind to a given virus.

As a further test, all 6179 experimental values are used in the regression model including the “greater than” approximate values. For these, the maximum experimental value is used; e.g. if the IC_50_ is expressed as >10 μg/ml and >50 μg/ml in two different studies, the value 50 μg/ml is used. The obtained regression model shows an increase in accuracy, in particular an increase ability in ranking different antibodies (Spearman correlation coefficient increases from 0.766 to 0.960), but it has a loss in sensitivity for all low-breadth antibodies predicting zero breadth for most antibodies with experimental breadth less than 0.4; see Fig 4 (center.) As a result, although the accuracy of the slope is improved, the regression line is shifted to the right, yielding worse values.

### Neural network classifier for the IC_50_

The breadth can be estimated more directly with an MLP classifier, which is trained to predict whether the IC_50_ is less or greater than the 1 μg/ml cutoff. A higher accuracy in the correlation between experiments and predictions is expected, because a classification is a simpler problem than a regression. The methodology used for the MLP classifier is the same as that employed for the MLP regressor, except that the whole set of 6179 experimental IC_50_ values is used; i.e., the 2351 “approximate” values are included. Table 1 lists the antibodies and the number of “exact” and “approximate” IC_50_ values available for each.

The correlation between the experimental and the computed breadth using the IC_50_ classifier is shown in Fig 4 (right). As expected, the results are significantly better than for the MLP regressor, with the correlation with the experimental breadth increasing from 0.800 for the regressor to 0.973 for the classifier. Moreover, no outliers are present and the estimated breadths of all 24 antibodies lie on the diagonal with near unit slope.

The performance of the MLP classifier is evaluated further by the analysis of its confusion matrix, which contains the percentage of true negative, true positive, false negative and false positive values. As reported in Table 2, the accuracy in the test set, corresponding to the sum of true positives and true negatives, is 72.3±1.0, with an almost equal rate of false positive and false negative of about 13%.

**Table 2:** Confusion matrix for the IC_50_ classifier. The accuracy (ACC) is defined as the sum of the true positive (TP) and true negative (TN): ACC = TP+TN; the balanced accuracy (BACC) is defined as the average between the true positive divided by total positive samples (P) and true negative divided by total negative (N) samples: BACC = (TP/P + TN/N)/2.

	True Negative	False Negative	False Positive	True Positive	Accuracy	Balanced Accuracy
Training set:	48.1±1.0	9.6±0.8	8.8±0.9	33.5±1.0	81.6±0.6	81.1±0.7
Validation set:	43.3±1.0	14.0±1.0	13.6±1.1	29.2±0.9	72.5±0.9	71.9±0.9

### Test of MLP classifier on germline or immature antibodies

Although our primary purpose for developing the MLP classifier is to evaluate antibodies designed to have increasing breadth (see Concluding Discussion), we decided to determine whether it could be used to compute the breadth of putative germlines, as compared to the breadth of the mature antibodies. We consider bnAbs VRC01, NIH45-46 and 3BNC60, for which the experimental breadths of the mature antibodies are available, see Table 3. For the putative germlines no experimental breadths are available, but the expected breadth should be low, as they appears to have no affinity for native HIV antigens [13–15].

**Table 3:** Comparison between calculated and experimental breadths for mature and germline forms of three antibodies. The experimental breadth of the germline antibodies is assumed to be zero (no quantitative data are available). The intermediate VRC01 antibody is DRVIA7.

Antibody	Breadth					
	Germline		Intermediate		Mature	
	Exp.	Calc.	Exp.	Calc.	Exp.	Calc.
VRC01	(0)	0.45±0.23	0.23	0.45±0.17	0.70	0.79±0.08
NIH45-46	(0)	0.20±0.17			0.77	0.81±0.06
3BNC60	(0)	0.27±0.23			0.78	0.53±0.21

The computed breadth values are reported in Table 3. For the mature antibodies the computed values are in good agreement with the experimental data. This is particularly interesting for the 3BNC60 antibody, for which no published structure of any antibody/antigen complex is currently available. For this reason, 3BNC60 was excluded from the training of the MLP regressor and classifier. However, a bound crystallographic structure is available for its putative germline precursor [16]. Assuming no significant change in the binding pose upon affinity maturation, the germline crystallographic structure was used for both the mature and germline antibody. This is likely to be a valid assumption, as indicated by the VRC01 and NIH45-46 antibodies for which crystallographic structures are available for both mature and germline forms bound to an antigen (see PDB 4JPK and 3NGB for VRC01 and 5IGX and 3U7Y for NIH45-46). The breadth of antibody 3BNC60 is slightly underestimated (0.53±0.21 vs 0.78), but it also has a higher than usual uncertainty.

For the putative germlines, the calculated breadths range between 0.27 and 0.45. Importantly, in all cases the breadth of the germline is significantly lower than the breadth of the mature antibody. This finding is encouraging, because only mature antibodies were used in the training of the MLP classifier, as no quantitative information for germline binding to HIV viruses is available in the CATNAP database. The MLP classifier is thus biased towards mature antibodies.

Finally, we consider the DRVIA7 antibody, which is an immature form of a VRC01-class antibody [17]. Few experimental IC_50_ values are available for this antibody; they show a breadth of about 0.23. The estimated value by modeling is 0.45±0.17, which is again higher than expected, but lower than the VRC01 mature value (0.79±0.08) and similar to the germline (0.45±0.23).

### Analysis of descriptors used in the MLP classifier

An important aspect in the present application of machine learning for the prediction of antibody breadth is the choice of descriptors. The 24 descriptors considered can be broadly divided into four categories: atomic descriptors such as buried surface area or number of hydrogen bonds (15 descriptors in total), protein/protein binding affinity measures (4 descriptors: Prodigy, ZRANK, ZRANK2 and DFIRE), protein folding scoring functions (FoldX and two pairwise statistical potentials: RFHA and RFCB), and entropy models (normal mode entropy from two different elastic network models: ENM_EXP and ENM_R6); see Methods for more details. Their relative correlation coefficients and their correlation with the experimental values are reported in Fig 3. As expected, some descriptors are highly correlated with each other, such as the number of hydrogen bonds evaluated using different definitions, or the van der Waals energy with the buried solvent accessible surface area.

Selecting too few descriptors would risk making the model less general. Consequently, we kept a large number of descriptors, using as discriminant the computational cost needed to evaluate them. From the full set of 24 descriptors, 5 are initially removed due to their computational cost. The remaining 19 descriptors are all very quick to evaluate, about 20 seconds in total for one model, obtaining a 12.8x speedup in the calculation of the descriptors with respect to whole set of 24 descriptors. No decrease in accuracy is observed.

If too many descriptors are used, there is risk of overfitting. This is avoided here by the very large number of available experimental values. For the MLP classifier 6179 experimental values are available, half of which are used in the fitting. The MLP classifier with 19 descriptors and 10 hidden nodes contains 200 fitting parameters (see Methods), versus the 3089 (6179/2) experimental values used in the fitting. A ratio between the number of experimental values in the training and number of parameters in the model of ten or greater is suggested [11]; here the ratio is >15. As supporting evidence, Table 2 shows that the accuracy of the MLP classifier in the training and test sets is similar.

**Fig 5:**
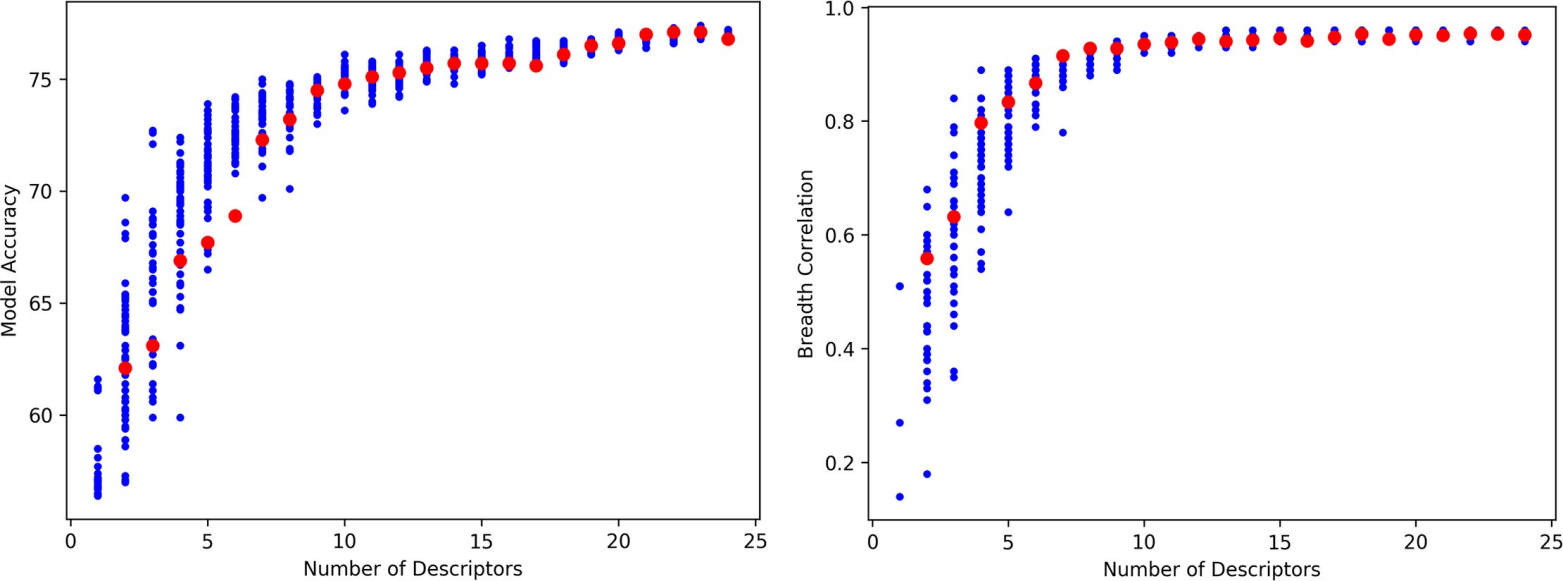
Accuracy (left) and correlation with the experimental breadth (right) obtained for MLP classifier trained with different sets of descriptors. On the abscissa is the number of descriptors used in each model. Blue points represent randomly generated models (selecting randomly between 1 and 24 descriptors among the 24 available), red points represent models obtained by systematic reduction of the full 19-descriptors model by successive removal of the most correlated descriptor (the 5 most computationally expensive descriptors are removed first to bring from 24 to 19 descriptors, and then the most correlated are removed).

We also studied the effects of the number of descriptors used in the MLP classifier on the accuracy of the model as measured by the confusion matrix; see Fig 5. Each sample in these two plots is a different MLP classifier model fitted over a different set of descriptors. The first graph shows the accuracy of the given model, as measured by the confusion matrix as sum of the percentages of true positive and true negative, while the second one shows the Pearson correlation coefficients obtained when comparing the calculated and experimental breadths. The blue points correspond to models with randomly-chosen descriptors, while the red points represent models obtained by successively removing the descriptor that is most correlated with all other descriptors and less correlated with the experimental IC_50_ (see Methods). The accuracy starts to decrease significantly when the number of descriptors is less than about 10. The Pearson coefficient is more robust and starts decreasing at around 7 descriptors. At this point, the maximum cross-correlation (Pearson coefficient) between the descriptors is about 0.4. These data suggest that a simpler model of about 7–10 descriptors could be used, which would have (approximately) the same accuracy of the 19-descriptors model; see Table 4. As expected from the earlier analysis, the breadth results converge more rapidly than the accuracy.

**Table 4:** Successive simplification of the 19-descriptors neural network model by elimination of the highest correlated descriptors. The first column contains the number of descriptors in the current model. Columns two and three list the two descriptors with the highest pairwise correlation coefficient, which is reported in the fourth column. The correlations with the experimental IC_50_ values are reported next. The last column reports the descriptor which is deleted.

No. of descriptors	Highest correlation between		Correlation	Correlations with experiments are		Descriptor deleted
19	MD_h1	MD_h2	0.964	0.028	0.057	MD_h1
18	ZRANK	ZRANKr	0.845	0.022	0.175	ZRANK
17	ENM_EXP	ENM_R6	0.833	0.292	0.239	ENM_R6
16	ZRANKr	DFIRE	0.731	0.175	0.044	DFIRE
15	IC_CP	RFCB	0.675	0.185	0.242	IC_CP
14	RFHA	ZRANKr	0.642	0.286	0.175	ZRANKr
13	MD_h2	IC_CC	0.632	0.057	0.041	IC_CC
12	MD_sasa	ENM_EXP	-0.590	0.094	0.292	MD_sasa
11	RFCB	RFHA	0.573	0.242	0.286	RFCB
10	IC_AP	IC_AC	-0.549	0.158	0.124	IC_AC
9	IC_AA	RFHA	-0.488	0.087	0.286	IC_AA
8	IC_PP	ENM_EXP	0.426	0.196	0.292	IC_PP
7	RFHA	NIS_A	0.407	0.286	0.116	NIS_A
6	ENM_EXP	RFHA	0.401	0.292	0.286	RFHA
5	ENM_EXP	NIS_C	-0.185	0.292	0.056	NIS_C
4	MD_h2	MD_h3	-0.154	0.057	0.084	MD_h2
3	IC_AP	ENM_EXP	0.113	0.158	0.292	IC_AP
2	MD_h3	ENM_EXP	0.028	0.084	0.292	MD_h3

This raises the question of which descriptors are most important in the prediction of the neutralization ability of an antibody. There are a variety of methods to extract descriptor importance from an artificial neural network, but there is no apparent consensus on which is better, in particular if the descriptors show a relative high level of cross correlation [18]. However, it is possible to extract some information from the correlation of the descriptors with the experimental IC_50_ values and with the deletion order reported in Table 4. The protein/protein scoring functions are on average more important than molecular descriptors, in particular the protein folding propensity descriptors (FoldX) and the two statistical pairwise potentials for estimating protein stability (RFHA and RFCB). Also, the entropy from elastic network models (ENM_EXP and ENM_R6) plays an important role, with ENM_EXP the last deleted descriptors with a relatively high correlation with the experiments (0.292 correlation coefficient). These highlight the importance of entropy in the binding, and the usefulness of complex protein potentials, with respect to simple molecular descriptors.

### MLP classifier generalization ability

**Fig 6:**
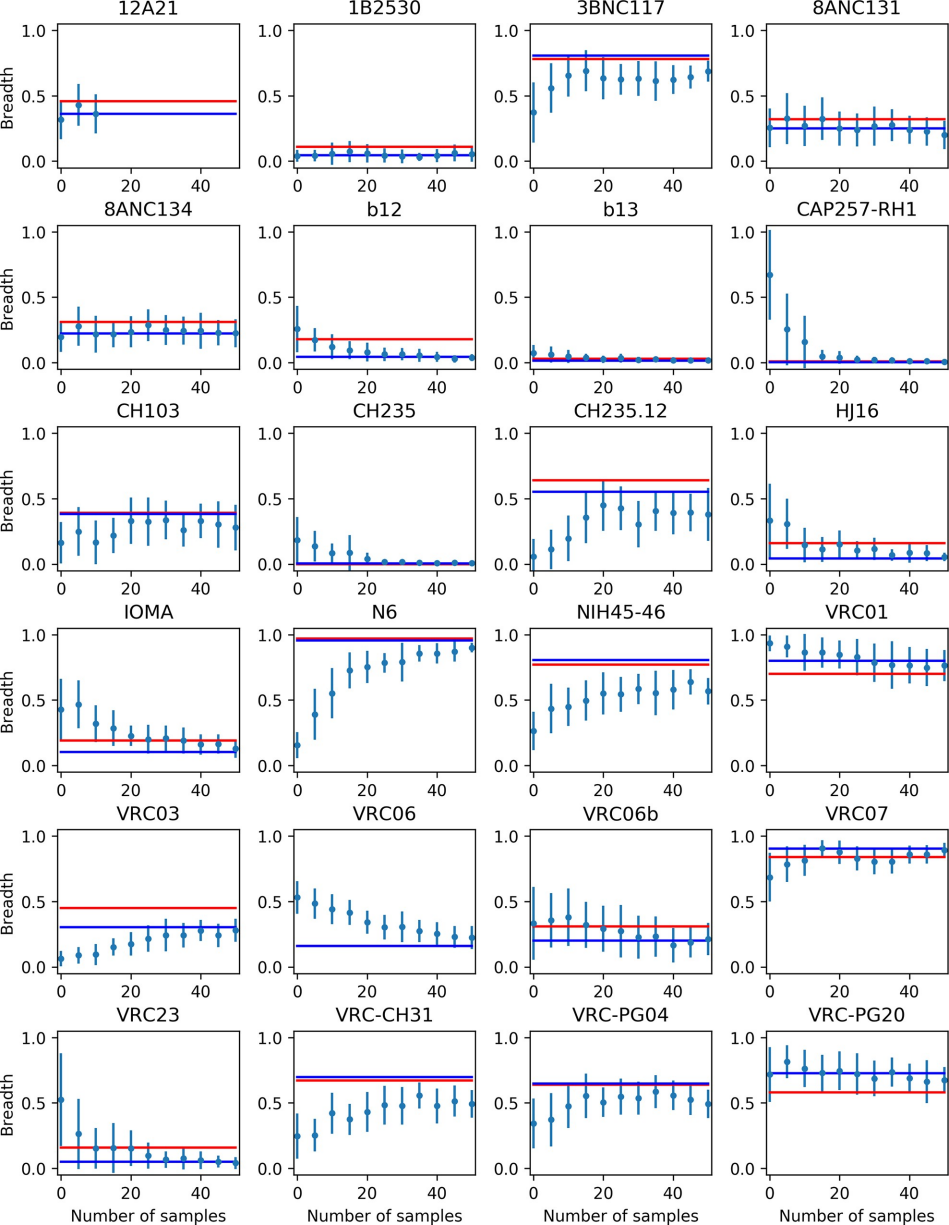
Computed breadth for the 24 antibodies studied in this work as a function of the number of experimental IC_50_ values for that particular antibody used in the training of the MLP classifier. The blue line is the computed breadth using all available experimental data in the training set, the red line is the experimental breadth. The graphs are shown up to 50 included experimental data, the total number of available experimental data is reported in Table 1.

An issue in machine learning models is how good they are at generalizing to inputs outside their training set [11,19,20]. In computational studies about HIV neutralization epitopes it is relatively common to have one model trained for each antibody under study [21–23]. By contrast, in this work only one model was trained to predict the neutralization breadth of all antibodies. We note that our training set did contain some experimental data from each antibody under study. This suggests the generalization ability of the model should be examined. Fig 6 shows how the predicted breadth of each antibody changes when an increasing number of experimental data relative to that antibody are included in the training set. At zero, no relevant experimental data are included; e.g. for the VRC01 antibody, at zero no experimental IC_50_ value of the VRC01 antibody is included in the training set. In these plots, the blue line is the breath computed using all available experimental data in the training set, while the red line is the experimental neutralization breadth. Comparing the plots for different antibodies, about half of them have a good breadth prediction even at zero, and the predicted breadth does not change significantly upon inclusion of more data; see for examples antibodies 1B2530, 8ANC131, 8ANC134, CH103, VRC01, VRC07, VRC-PG20. For other antibodies the prediction at zero is quite far from the experimental value; e.g. antibodies NIH45-46, CAP257-RH1, CH235.12, or N6. However, all these antibodies converge to the correct experimental value when a relatively small number of experimental values are included in the training set, usually between 10 and 20, relative to the total number of values available (see Table 1). The Pearson correlation coefficient between the computed and experimental breadth is 0.50 for the classifier with zero experimental data when all but 4 out of the 23 antibodies studied are included in the analysis (excluded antibodies are N6, CAP257-RH1, CH235.12 and NIH45-46, which have the highest error in the computed breadth). When the entire set of antibodies is considered, the correlation coefficient is 0.14; including just one experimental value per antibody, the Pearson coefficient increases to 0.54, and it reaches 0.90 when including only four experimental values. Another point to consider is that the good results obtained for some antibodies are due to their close similarity to other antibodies in the training set; e.g. the 8ANC131/8ANC134 pair and the VRC01/VRC07 pair. These results show that the neural network model is able to predict accurate values for the breadth in instances when a small number of experimental data for the antibody under study, or a close relative, are included in the training set.

### Comparison with other machine learning techniques

It is of interest to compare the results from MLP classifier used here with other machine learning techniques. We compared the Neural Network with one hidden layer used in this paper with a Neural Network with two hidden layers, k-nearest neighbors (kNN) [24], random forests (RF) [25,26], and Support Vector Machine (SVM) [27]; see Methods. All methods produce very similar results, in both the correlation with the experimental breadths and the estimated error in the single breadth values; see Table 5. This is supporting evidence of the robustness of the results obtained with the MLP. Moreover, it suggests that to improve the results new information about the system needs to be introduced, e.g. in the form of different descriptors.

**Table 5:** Comparison of the MLP classifier results with other machine learning techniques: MLP classifier with two hidden layers, Random Forests (RF with 31 trees), Supported Vector Machine (SVM with radial basis function kernel), and k-nearest neighbors (with 15 neighbors and weights assigned inversely proportional to the distance). The reported data are the accuracy of the classifier (from the confusion matrix), and the Pearson, Spearman, slope and intercept for the correlation of the calculated and experimental breadths.

Method	Accuracy	Breadth correlation			
		rp	rs	Slope	Intercept
MLP classifier (1 hidden layer)	72.5±0.9	0.973	0.979	1.11	-0.079
MLP classifier (2 hidden layers)	72.5±0.8	0.969	0.979	1.167	-0.081
Random Forest (31)	75.5±0.6	0.969	0.973	1.151	-0.104
SVM (rbf)	76.0±0.6	0.909	0.935	1.424	-0.216
K-nearest neighbors (15, distance)	75.5±0.7	0.979	0.984	1.225	-0.097

## Discussion

To the best of our knowledge, we report in this paper the first attempt to estimate the breadth of HIV antibodies by the use atomistic modeling and machine learning techniques. We developed a method based on a Multi-Layer Perceptron (an artificial neural network), which is able to accurately reproduce and predict the breadth of CD4bs targeting HIV antibodies.

For the development of such a model, many experimental IC_50_ values to train the neural networks have to be available. Here we used experimental IC_50_ values from the CATNAP database [12]. After cleaning and filtering the data, 6179 IC_50_ values were obtained for the training of the classifier and 3864 for the regressor. These significantly outnumber the number of parameters in the neural network (200), reducing the possibility of overfitting. For best results, a small number of experimental values specific for the antibody under study, or a related antibody, need to be present in the training set. For the application of the same techniques to different HIV binding sites or different viruses, such as influenza, sufficient high-quality experimental data would have to be collected and validated.

An important concern in any HIV model is the glycosylation of the virus since the HIV Envelope protein is heavily glycosylated. In the CD4bs, the focus of this work, there are at least five glycans (N197, N234, N276, N363, and N462) that can interact, and interfere, with the antibody binding. One glycan, N276, is particularly problematic in a comparison between germline and mature antibodies, as it has been observed that mature antibodies, like VRC01, introduce deletions or mutations to glycine in CDRL1 to avoid clashes and to accommodate the glycan [28,29]. This could be a major reason why the current model overestimates the breadth of putative germlines.

The tools presented here are important for the computational design and screening of potential HIV antibodies. The usual focus in antibody design is in the optimization of the potency and specificity for a particular antigen [30]. As pointed out in the Introduction, this is only one part of the problem for highly mutable virus like HIV, for which the exposed antigens have a high variability, so that bnAbs are required. Thus, it is important to couple the calculation of the potency with an estimation of the breadth.

The present technique for estimating antibody breadth based on IC_50_ values could prove useful in the computational design of vaccination protocols to elicit HIV bnAbs. Promising computational/theoretical approaches for the design of vaccination strategies include a description of the affinity maturation (AM) process [31,32]. This is achieved by simulating how antibodies mutate during the AM from the germline precursors to the mature antibodies. One essential step in these simulations is evaluating the breadth of the produced antibodies. Since the method described here requires as inputs only the sequence of the antibody and a template for the bound structure, it is likely to be a useful part of the design process. Moreover, the techniques developed here can, in principle, be applied to other highly variables viruses for which a definition of antibody breadth is important, such as influenza or hepatitis C viruses, if sufficient experimental neutralization data are available for training the models. From preliminary examination of the literature, this appears to be true at least for influenza.

An alternative perspective is to consider the evaluation of the breadth from the antibody/antigen binding affinity, see S1 Appendix. This requires an accurate, relatively rapid, method for the calculation of the binding affinity. Research on developing such a methodology is in progress [33]. The use of the “free energy of binding breadth”, rather than the “IC_50_ neutralizing breadth”, would, in principle, bypass the need for machine learning techniques.

## Methods

First, the source of experimental IC_50_ values used in the training of the neural network is described. Then, the procedure to create an atomistic model of antibody/antigen complexes is presented, followed by the computation of all molecular descriptors derived from the atomistic models. The computational cost of creating the models and evaluating the descriptors is then reported. The final section is dedicated to the details of the neural network models.

### Experimental IC_50_ database

To calculate the breadth of an antibody, the IC_50_ values for the antigens in the virus panel are required. In this work the experimental IC_50_ are used to both calculate the experimental breadth and to train a neural network (see below) to predict the IC_50_ values and breadth of new antibodies.

Experimental values of the IC_50_ for different antibody/antigen complexes were obtained from the CATNAP database [12] (accessed on March 5, 2018). The IC_50_ values are presented in the database in two forms. If available, the exact value is reported; if the complex has a low binding affinity, the exact value is not available, and the IC_50_ is reported to be greater than a given cutoff, e.g. “IC_50_>50μg/ml”. The full database contains information for 1024 HIV viruses and 334 antibodies, for a total of 23851 exact and 16467 approximate IC_50_ values (most antibodies are tested on different viruses). Of interest in this work are antibodies binding on the CD4bs. Because our method requires atomistic models of the antibody/antigen complex, IC_50_ values are selected such that the binding is at the CD4bs (based on known crystallographic structures of the bound antibody/antigen complex), the amino acid sequences of both the antibody and the antigen are known, and at least one crystallographic structure of the complex is experimentally available. After applying these filters, 24 antibodies (over the 334 available) are selected, for which a total of 3864 exact and 2315 approximate IC_50_ values are available; see Table 1.

### Creating 3D atomistic models

The protocol described in this work requires building of atomistic models for arbitrary antibody/antigen complexes and computing molecular descriptors to train the neural network models. The first task is to build a full 3D atomistic model of the antibody/antigen complex given only the sequences of the two proteins. A requirement is to have a template of the complex, which is used for homology modeling. We select one template for each antibody, neglecting differences in the antigens. The underlying assumption is that the binding mode of a given antibody to any antigen is very similar and can be approximated to be constant. Moreover, the structure of the antigen is assumed to be conserved over the sequence space. This is not generally true for antigens with significant insertion or deletions in their (hyper)variable loops. Because we focus on the CD4bs, the assumption is reasonable. Crystallographic structures of antibody/antigen complexes were used as templates for the 24 antibodies. In most cases, the antibody was not bound to naturally-occurring antigens, but to engineered monomeric (gp120) domains. In these cases, the engineered protein was substituted with the BG505 SOSIP structure (PDB 5FYJ) using best-fit RMSD alignment for the superimposition. The PDB codes used for each antibody are reported in Table 1.

Given the sequences of the antigen and the antibody and a template crystallographic structure for the complex, Modeller [34] was used to create a model of the complex. The structure was then refined by CHARMM [35], including adding missing hydrogen atoms, creating disulfide bonds, and minimizing the all-atom structure by 100 steps steepest descent. The system was then further minimized using OpenMM [36] until the potential energy was changing by less than 1 KJ/mol. The CHARMM36 force field [37] was used for the all-atoms energy minimizations. In the modeling, only one monomer of the Env gp160 trimer was built, the gp41 deleted, and only the variable part of the antibody was kept. Glycosylation was not included.

### Molecular descriptors

The optimized structure obtained by molecular modeling (see previous paragraph) was used for the computation of all descriptors to train the neural networks. 24 molecular descriptors were computed. These are divided in four main classes: atomic descriptors, protein/protein binding scoring functions, protein stability scoring, and entropy models.

As “atomic descriptors” we consider descriptors that are simple function of the atomic coordinates. The two most obvious are the electrostatic (Coulomb) interaction between the antibody and the antigen (E_elec) and the dispersive (Lennard-Jones) interaction (E_vdw). These are modeled according to the CHARMM36 force field [37] and computed using OpenMM [36]. To approximate the solvation of the protein, the GBn2 [38,39] generalized Born implicit model was used as third descriptor (E_gbsa). No cutoff in either electrostatic or dispersion force was used. Other used descriptors are the buried surface area [40] (MD_sasa) upon binding, the number of hydrogen bonds according to Baker & Hubbard [41] (MD_h1) and according to Wernet, Nilsson *et al*. [42] (MD_h2) definitions, and hydrogen bond energy according to Kabsch & Sander [43] (MD_h3). These four descriptors were computed using the MDTraj [44] python module. To these, the number of interchain contacts classified according to polarity and charge are added (a total of six descriptors: IC_AA, IC_PP, IC_CC, IC_AP, IC_CP, IC_AC), as well as the apolar and charged non-interacting surface area (NIS_A and NIS_C). These last eight descriptors were computed using Prodigy [45].

More complex descriptors are scoring functions developed for estimating the binding affinity of two proteins. These methods have been developed to score binding modes in protein/protein docking software. Here, they are not used as independent scoring functions (as originally intended), but as descriptors. The used methods are: Prodigy [45], ZRANK [46], ZRANKr [47,48], and DFIRE [49].

The third class of descriptors consists of scoring functions optimized to reproduce the folding propensity of a protein or its stability. With these scoring functions, the binding affinity can be estimated as the difference between the “stability” of the complex and that of the separated antibody and antigen. The methods tested are: FoldX [50,51] (sidechains are optimized), and two pairwise statistical potentials (RF_HA_SRS and RF_CB_SRS_OD) [52,53].

The fourth class of descriptors contains two methods used to estimate the entropy change upon binding. Two classical approaches to evaluate the entropy of a macromolecule are the use of the normal mode analysis (NMA) or quasi-harmonic (QHA) [54]. Neither of these can be used here directly: NMA requires that the structure is at an energy minimum, and QHA requires a large ensemble of structures. Alternatives are Elastic Network Models (ENM) [55,56]. In these models, the protein is modeled as a set of atoms interconnected by elastic springs, which vibrate around the given input structure. The total energy *E* of the system is obtained as sum of pairwise potentials, each acting between a pair of atoms whose distance is under a given cutoff: *E* = ∑_*ij*_*k*(*d*_0_)∙(*d_ij_*−*d*_0_)^2^. The sum is over all pairs of atoms *ij*, *d_ij_* is the distance between them, *d*_0_ is the reference distance (from the input structure), and *k*(*d*_0_) is the force constant. *k*(*d*_0_) can take different expressions. The first developed used a constant for each pairs under a given distance cutoff [55]. More accurate models use force constants that depend on the reference distance *d*_0_. In this paper two expressions are tested. In the first (ENM_EXP) the force constant decreases exponentially with *d*_0_: $k(d_{0})=aexp\left[{\left(\frac{d_{0}}{r}\right)}^{2}\right]$, with *r* = 7Å [57]. In the second (ENM_R6), the force constant decreases proportionally to $d_{0}^{-6}:\;k(d_{0})=ad_{0}^{-6}$ [58]. In both cases an arbitrary proportionality constant *a* has to be set. Here the value of 1000 is used (in arbitrary units). A 100-fold increase in the force constant caused no significant change in the results. From the energy expression, the Hessian matrix can be calculated, and, upon diagonalization of the mass-weighted Hessian matrix, the normal frequencies are obtained, and from them the harmonic entropy. These calculations are fast and do not require energy minimization as the input structure is considered to be the minimum.

A problem with most of the descriptors used in this work is their high sensitivity to the values of the atomic coordinates. To alleviate this problem, six models for each complex are generated with Modeller using random initial seeds, and the descriptors are averaged over the six models.

### Timing

An important factor to consider when designing and choosing descriptors to use in neural networks and regressor models is the computational cost, or timing, needed to compute them. In this work, two are the main steps: first, generate the 3D atomistic model; second, compute all the descriptors.

As described above, preparation of the atomistic model consists of three steps. Modeller takes on average 69 seconds to generate one model. CHARMM, used to fix the structure and run a quick minimization takes an insignificant amount of time. A deeper energy minimization with OpenMM takes on average 37 seconds. A total of about 100 seconds are thus needed to generate one model.

The time required for each descriptor varies significantly. Most descriptors are computed almost instantaneously, requiring one second or less. There are three exceptions: evaluating the OpenMM-based descriptors (electrostatic, dispersive and generalized born energies) takes about 51 second per model, the ENM entropy takes 15 seconds, and FoldX is the slowest descriptor used, requiring 233 seconds on average. Computing all descriptors requires about 5 minutes. Skipping FoldX and OpenMM (the two most expensive steps) decreases the time from 5 minutes to 20 seconds.

These timings are for one single model. For each antibody/antigen complex six models are used, and the descriptors are averaged. This means that to create the six models 10.6 minutes are required, and to get all the descriptors, an additional 30.8 minutes are needed, for a total of 41 minutes. Without FoldX and OpenMM, the timing for the descriptors decreases to 2.4 minutes, reducing the total time to about 13 minutes.

### Neural network models

To predict the IC_50_ values a Multi-Layer Perceptron regressor (MLP regressor) is used. This type of artificial neural network takes as input a number of descriptors, parse them in a hidden layer composed, in this case, of ten nodes, and outputs the predicted IC_50_ value. For the training, the 3864 exact IC_50_ values are split randomly in half into a training set and a validation set. The total number of parameters N_p_ to fit in the MLP is given by N_p_ = N_i_N_h_+N_h_N_o_ where N_i_, N_h_, and N_o_ are the number of input descriptors, hidden nodes, and outputs. Using 19 descriptors, and requiring one output (the IC_50_ value), the total number of parameters to fit is 200. As an empirical rule, the ratio between the available experimental values used in the training and the number of parameters to fit has to be much greater than one. In our case, the number of experimental IC_50_ values used for the training is 3864/2, which is 9 times higher than the number of parameters to fit. A logistic activation function is used in the hidden nodes, and the lbfgs solver is used for optimization. Fig 3 shows the cross correlation between all descriptors and the experimental IC_50_ values.

A similar artificial neural network is used to predict whether the IC_50_ is less or greater than the experimentally-derived 1 μg/ml cutoff, which is used to calculate the antibody breadth. In this case, a Multi-Layer Perceptron classifier (MLP classifier) is trained instead of a regressor on the same set of descriptors. The same settings as for the MLP regressor were used, with the only difference that the output is filtered with a logistic function to be between 0 and 1. Here the total number of experimental values is 6179. Of these, 3089 are randomly selected and used to train the model. The ratio between the number of experimental values in the training (3089) and the number of parameters (200) is more than 15, decreasing the chance of overfitting. Table 6 compares the composition of the full dataset and a randomly generated set containing 50% of the values. All antibodies are represented in the training set, with the same percentages of binders and not-binders as in the whole set.

**Table 6:** Composition of the IC_50_ database used to train the IC_50_ classifier. One set of data is available for each antibody: the line “All” indicates the statistics for the whole data set, “Random” for a randomly generated set of containing 50% of the data. Count is the number of complexes containing that antibody, with percentages relative to the full data set. “Bind” and “Not bind” are the numbers (percentages) of complexes for which binding and no binding is detected.

Antibody	Data set	Count (%)	Bind (%)	Not bind (%)
VRC01	All	609 (9.9%)	363 (59.6%)	246 (40.4%)
	Random	297 (9.6%)	172 (57.9%)	125 (42.1%)
b12	All	690 (11.2%)	81 (11.7%)	609 (88.3%)
	Random	362 (11.7%)	37 (10.2%)	325 (89.8%)
VRC03	All	318 (5.1%)	111 (34.9%)	207 (65.1%)
	Random	159 (5.1%)	47 (29.6%)	112 (70.4%)
N6	All	350 (5.7%)	332 (94.9%)	18 (5.1%)
	Random	186 (6.0%)	179 (96.2%)	7 (3.8%)
3BNC117	All	444 (7.2%)	300 (67.6%)	144 (32.4%)
	Random	192 (6.2%)	135 (70.3%)	57 (29.7%)
b13	All	176 (2.8%)	4 (2.3%)	172 (97.7%)
	Random	79 (2.6%)	2 (2.5%)	77 (97.5%)
NIH45-46	All	265 (4.3%)	193 (72.8%)	72 (27.2%)
	Random	127 (4.1%)	100 (78.7%)	27 (21.3%)
VRC07	All	389 (6.3%)	312 (80.2%)	77 (19.8%)
	Random	197 (6.4%)	150 (76.1%)	47 (23.9%)
VRC-CH31	All	265 (4.3%)	167 (63.0%)	98 (37.0%)
	Random	117 (3.8%)	75 (64.1%)	42 (35.9%)
VRC-PG04	All	285 (4.6%)	173 (60.7%)	112 (39.3%)
	Random	139 (4.5%)	82 (59.0%)	57 (41.0%)
CH103	All	191 (3.1%)	86 (45.0%)	105 (55.0%)
	Random	103 (3.3%)	49 (47.6%)	54 (52.4%)
CH235	All	196 (3.2%)	2 (1.0%)	194 (99.0%)
	Random	105 (3.4%)	0 (0.0%)	105 (100.0%)
CH235.12	All	194 (3.1%)	112 (57.7%)	82 (42.3%)
	Random	109 (3.5%)	61 (56.0%)	48 (44.0%)
IOMA	All	117 (1.9%)	17 (14.5%)	100 (85.5%)
	Random	64 (2.1%)	9 (14.1%)	55 (85.9%)
1B2530	All	171 (2.8%)	29 (17.0%)	142 (83.0%)
	Random	80 (2.6%)	9 (11.2%)	71 (88.8%)
8ANC131	All	178 (2.9%)	52 (29.2%)	126 (70.8%)
	Random	89 (2.9%)	27 (30.3%)	62 (69.7%)
8ANC134	All	169 (2.7%)	48 (28.4%)	121 (71.6%)
	Random	90 (2.9%)	27 (30.0%)	63 (70.0%)
CAP257-RH1	All	191 (3.1%)	1 (0.5%)	190 (99.5%)
	Random	91 (2.9%)	1 (1.1%)	90 (98.9%)
HJ16	All	252 (4.1%)	39 (15.5%)	213 (84.5%)
	Random	133 (4.3%)	23 (17.3%)	110 (82.7%)
VRC06	All	176 (2.8%)	24 (13.6%)	152 (86.4%)
	Random	95 (3.1%)	11 (11.6%)	84 (88.4%)
VRC06b	All	174 (2.8%)	51 (29.3%)	123 (70.7%)
	Random	93 (3.0%)	24 (25.8%)	69 (74.2%)
VRC23	All	175 (2.8%)	29 (16.6%)	146 (83.4%)
	Random	90 (2.9%)	16 (17.8%)	74 (82.2%)
VRC-PG20	All	178 (2.9%)	113 (63.5%)	65 (36.5%)
	Random	79 (2.6%)	56 (70.9%)	23 (29.1%)
12A21	All	26 (0.4%)	11 (42.3%)	15 (57.7%)
	Random	13 (0.4%)	4 (30.8%)	9 (69.2%)
Total	All	6179	2650 (42.9%)	3529 (57.1%)
	Random	3089	1296 (42.0%)	1793 (58.0%)

It was observed that the outputs of both the MLP regressor and MLP classifier would change, in some cases significantly, upon repeated training using different random splitting of the experimental values into training and validation sets, and upon fitting the neural networks using different random seeds. For this reason, we average over 30 independently generated neural networks using each time a new splitting of the training and validation set and a new random seed in the training. The standard deviation between the 30 replicas is used as an estimate of the statistical error.

To select which descriptors to use in the neural network, the first criterium was to keep the scoring function as fast as possible, avoiding the more computationally expensive OpenMM energy-based terms and FoldX. Removing these four descriptors does not cause a decrease in accuracy, while reducing the required time by a factor of 12.8. Prodigy was also removed, as it is a linear combination of other descriptors (no effect is observed in the accuracy after removal). This way, the number of descriptors used decreases from 24 to 19 without loss in accuracy. To study the performance of simplified models including fewer descriptors, the models are sequentially simplified by removing the highest correlated descriptor. To do this, the cross-correlation matrix with all descriptors is analyzed to find the pair of descriptors with the highest correlation among themselves; see Fig 3. The descriptor with the lowest correlation with the experimental IC_50_ values is removed. The process is repeated until only one descriptor is present. The list of descriptors which are deleted at each step (and their correlation coefficients) are reported in Table 4. All descriptors are normalized to zero mean and unit variance (standard scaler).

To verify the robustness of the neural network, the same analyses were repeated using other machine learning methods: k-nearest neighbors (kNN) [24], random forests (RF) [25,26], and support vector machine (SVM) [27]. First, the MLP were compared with a second MLP containing two hidden layers instead of one. Having observed the same results, it was compared with a kNN (with distance weighting), a RF (composed of 31 trees) and a SVM (with radial basis function kernel) and no significant improvement was observed with any of them.

All machine learning methods (MLP, kNN, RF, SVM) and the standard scaler are used as implemented in the scikit-learn [59] python module.

## Supporting information

S1 Appendix Computation of the breadth from the antibody/antigen binding affinity. (PDF)

S1 Data Python scripts and data to reproduce the figures and tables in the text. (TGZ)
