# Supplementary material for: Estimation of the breadth of CD4bs targeting HIV antibodies by molecular modeling and machine learning
Source: PLoS Comput Biol. 2019 Apr 10;15(4):e1006954. doi: 10.1371/journal.pcbi.1006954 (PMC6457539; doi:10.1371/journal.pcbi.1006954)
Supplement: S1 Appendix — (PDF) [file pcbi.1006954.s001.pdf]

Estimation of the breadth of CD4bs targeting HIV antibodies by molecular modeling and machine learning

Supporting Information

Simone Conti[1,*] and Martin Karplus[1,2,*]

[1] Department of Chemistry and Chemical Biology, Harvard University,

02138 Cambridge, Massachusetts, United States

[2] Laboratoire de Chimie Biophysique, ISIS, Université de Strasbourg,

67000 Strasbourg, France

*Corresponding authors: SC sconti@fas.harvard.edu MK marci@tammy.harvard.edu

Breadth from Binding Affinities

An alternative to the use of IC_{50} for defining the breadth, would be to use the binding affinity of the antibody/antigen complex (the standard free energy of binding, ΔG). The main advantage is that ΔG values can be obtained by well-studied computational methods. By contrast, IC_{50} values depend on the experimental conditions of their evaluations, like the substrate concentration, and the binding mechanics and kinetics, which make a computational evaluation from first-principles difficult. It is for these reasons that the machine learning method was developed for the IC_{50}. The use of ΔG values would make it possible to move away from data–driven neural networks for computing the breadth to methods based on statistical thermodynamics and free energy calculations.

Assuming a rank correlation between the IC_{50} and the binding affinity, we can define a “binding breadth” based on the ΔG values, to complement the “neutralizing breadth” based on the IC_{50}. As for the

IC_{50} based neutralizing breadth, the binding breadth of an antibody can be defined as the fraction of antigens in a panel for which the binding affinity is more negative than a given free energy cutoff (E_{cutoff}), that is $\Delta G < E_{cutoff}$.

As an example, the binding affinity of a given antibody for a target antigen is here approximated by a simple scoring function determined from experimental ΔG values from the literature. A total of 107 binding affinity values have been collected from four references [1–4]. Most of the values are single amino acid mutations to alanine on a common antibody/antigen complex. Due to the limited number of experimentally available ΔG values (107 values), a neural network cannot be trained with them. Consequently, a simple linear regression of molecular descriptors is used. Different combinations of descriptors were tested, of which the main ones are shown in Table 1; for definition see the Main Text. The primary criterion for a scoring function is a good correlation with the experimental binding affinities. Second, the average binding affinity of a given antibody against a panel of antigens should be correlated with the breadth, such that antibodies with a more negative average binding affinity have a higher breadth.

The correlation coefficients for the tested scoring functions are reported in Table 1. The best method based on the RFHA binding score [5,6] only reaches a Pearson correlation coefficient with the experimental binding affinities of 0.57. Figure 1 shows the smoothed distribution of binding affinities for the 24 antibodies studied in this work with all the antigens in the Seaman panel. The color of each curve is proportional to the experimental breadth of the antibody: red is high breadth, while blue is low breadth. From the plot the mean of the binding affinity is proportional to the breadth, with most high breadth antibodies (in red) in the high binding affinity region (left in the plot) and the low breadth antibodies (blue) in the low affinity region (right). This makes it possible to define an energy cutoff at -9 kcal/mol, to calculate the binding breadth of each antibody. The correlation between the computed binding breadths and the experimental neutralization breadths is shown in Figure 2. The obtained

correlation is reasonable for most antibodies with a tendency towards overestimation. A few antibodies are significantly overestimated, like IOMA, b12 and VRC06, and one antibody, CH235.12, is significantly underestimated. Overall, the correlation yields a Pearson coefficient of 0.600 and Spearman of 0.668.

These results, which were obtained with a simple free energy scoring function, show that it is possible to define a free energy cutoff to calculate the breadth. The obtained "binding breadths" correlate with the experimental "neutralizing breadths", even if some significant outliers are present. If more accurate atomistic free energies for the binding affinity become available from calculations, it is likely that improved binding breadth results could be obtained.

Scoring Function	Number of parameters	ΔG exp vs calc		Optimal cutoff	IC50 vs ΔG		avg(E) vs Breadth		Breadth exp vs calc	
		rp	Slope		rp	slope	rp	slope	rp	slope
RFHA	1	0.57	0.30	-9.0	-0.45	-0.81	-0.63	-1.65	0.60	0.85
RFCB	1	0.49	0.28	-10.0	-0.33	-0.90	-0.37	-0.63	0.26	0.36
ZRANK	1	0.30	0.13	-8.5	0.12	0.33	0.10	0.17	0.32	0.01
ZRANKr	1	0.18	0.06	-10.5	0.33	0.77	0.41	0.86	-0.45	-0.56
Prodigy	1	0.38	0.16	-8.5	0.26	0.87	0.35	0.52	0.33	0.00
FoldX	1	0.24	0.06	-10.0	0.24	1.49	0.47	0.35	-0.50	-0.42
DFIRE	1	0.35	0.11	-9.0	0.06	0.27	0.24	0.27	0.32	0.01
IC+NIS	8	0.48	0.26	-8.0	-0.19	-0.31	-0.14	-0.44	0.27	0.29
Eomm	3	0.56	0.34	-9.5	-0.07	-0.10	-0.25	-0.53	0.31	0.28
Eomm+SASA	4	0.56	0.28	-9.0	-0.06	-0.10	-0.25	-0.45	0.30	0.24
RFHA+ENM	2	0.56	0.30	-8.5	-0.45	-0.69	-0.63	-1.92	0.59	0.80

Table 1: Tested linear regressions to reproduce the experimental binding affinities. In order are reported: the list of parameters in the linear regression and their number, the correlation with the experimental binding affinities, the optimal free energy cutoff to calculate the breadth, the correlation between the predicted binding affinities and the predicted IC_{50} values, the correlation between the average binding affinity and the breadth, and the correlation between the experimental neutralizing breadth and the computed binding breadth.

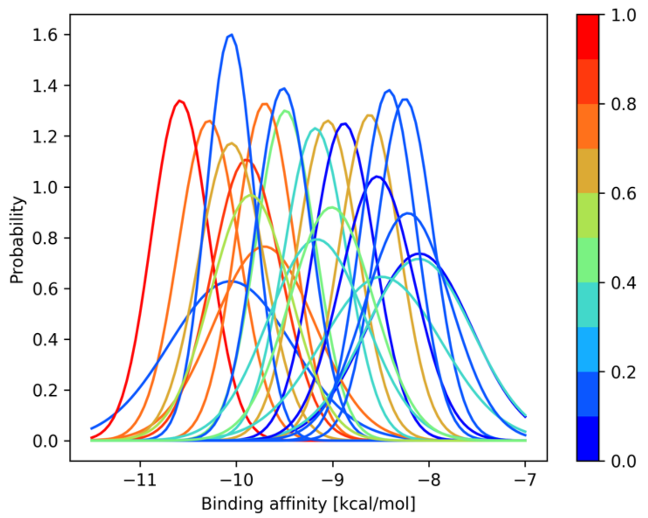

Figure 1: Smoothed histograms representing the distributions of binding affinity values for each of the 24 studied antibodies. The color correlates with the experimental breadth of that antibody (red: high breadth, blue: low breadth). High breadth antibodies have a high average binding affinity (left on the graph), while low breadth antibodies have a low high binding affinity (right).

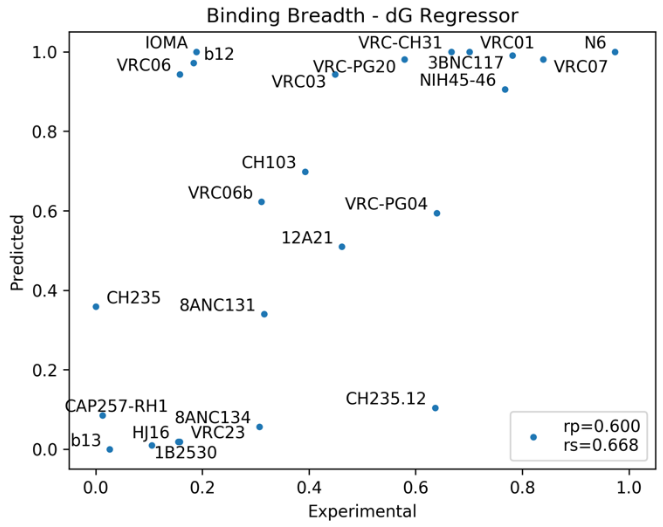

Figure 2: Correlation between the predicted binding breadth for 24 antibodies evaluated using the binding affinity values obtained by the RFHA scoring function and the experimental neutralizing breadths.

References

1. Zhou T, Georgiev I, Wu X, Yang Z-Y, Dai K, Finzi A, et al. Structural Basis for Broad and Potent Neutralization of HIV-1 by Antibody VRC01. Science. 2010;329(5993):811–7.

2. Clark AJ, Gindin T, Zhang B, Wang L, Abel R, Murret CS, et al. Free Energy Perturbation Calculation of Relative Binding Free Energy between Broadly Neutralizing Antibodies and the gp120 Glycoprotein of HIV-1. J Mol Biol. 2017;429(7):930–47.

3. Diskin R, Scheid JF, Marcovecchio PM, West AP, Klein F, Gao H, et al. Increasing the Potency and Breadth of an HIV Antibody by Using Structure-Based Rational Design. Science. 2011;334(6060):1289–93.

4. Zhou T, Xu L, Dey B, Hessell AJ, Van Ryk D, Xiang S-H, et al. Structural definition of a conserved neutralization epitope on HIV-1 gp120. Nature. 2007;445(7129):732–7.

5. Rykunov D, Fiser A. Effects of amino acid composition, finite size of proteins, and sparse statistics on distance-dependent statistical pair potentials. Proteins Struct Funct Bioinforma. 2007;67(3):559–68.

6. Rykunov D, Fiser A. New statistical potential for quality assessment of protein models and a survey of energy functions. BMC Bioinformatics. 2010;11:128.
