# Supplementary figures and images for: Estimation of the breadth of CD4bs targeting HIV antibodies by molecular modeling and machine learning
Source: PLoS Comput Biol. 2019 Apr 10;15(4):e1006954. doi: 10.1371/journal.pcbi.1006954 (PMC6457539; doi:10.1371/journal.pcbi.1006954)

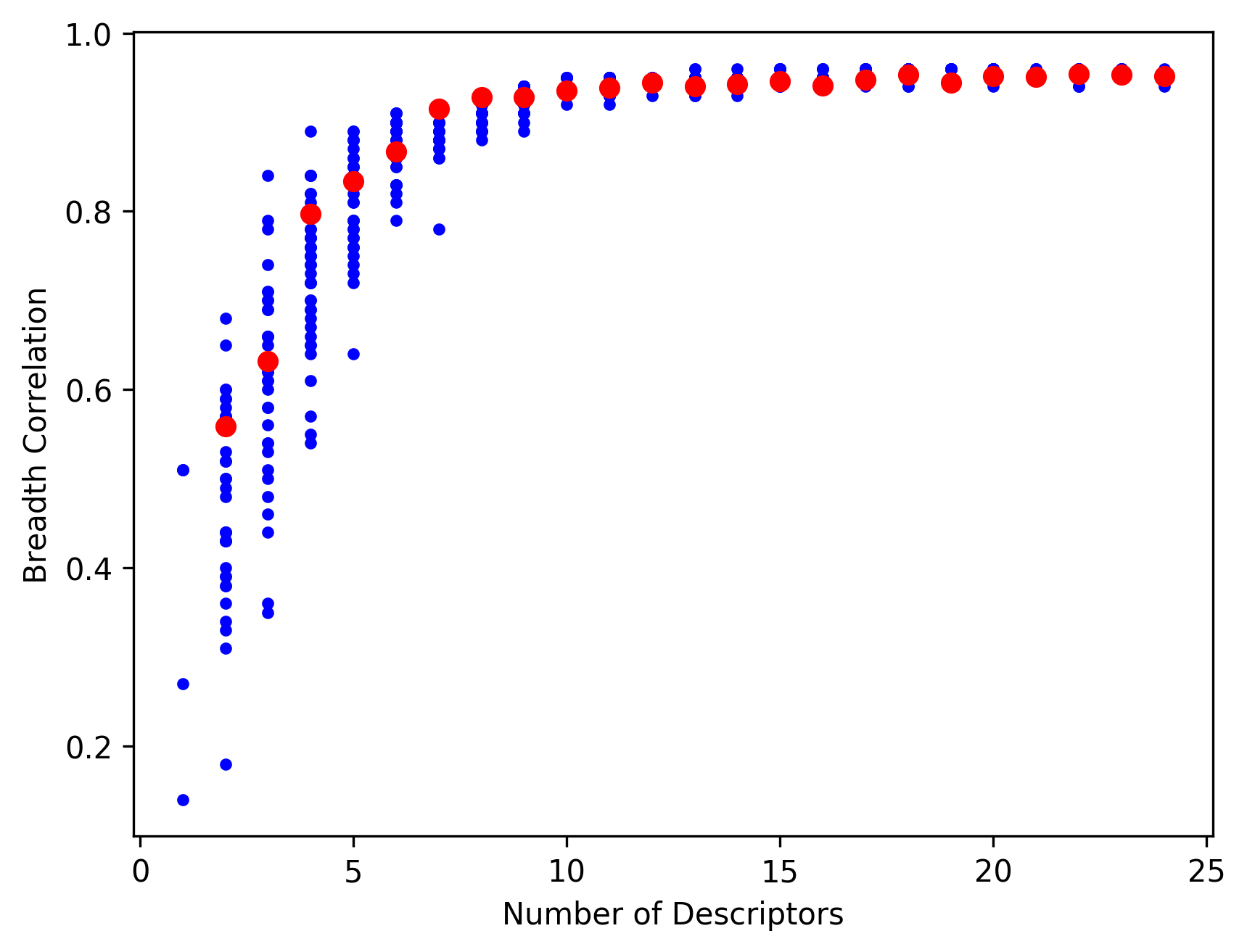

Supplement: S1 Data — (TGZ) [file pcbi.1006954.s002.tgz › supporting_data/sample_outputs/fig5_pearson.png]

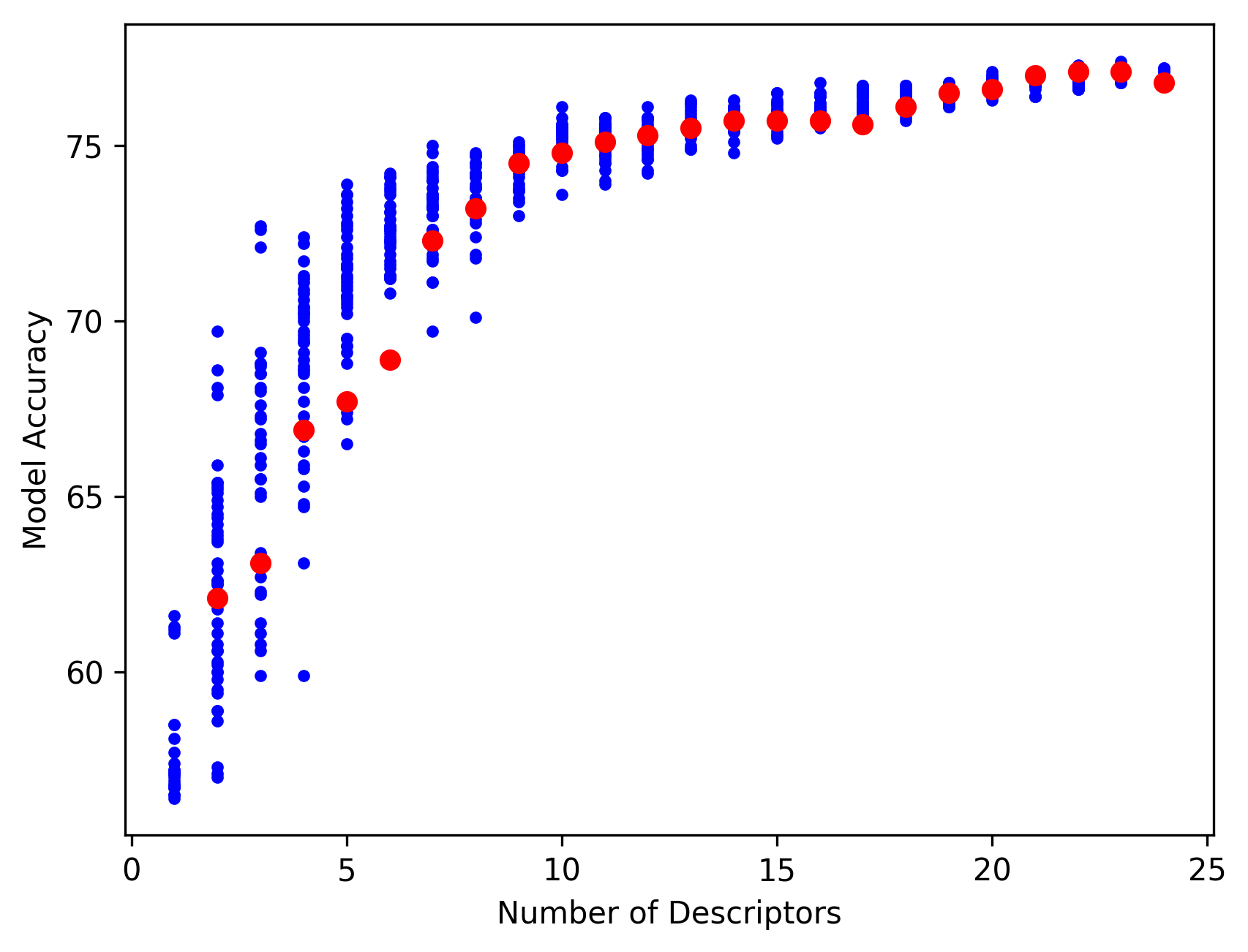

Supplement: S1 Data — (TGZ) [file pcbi.1006954.s002.tgz › supporting_data/sample_outputs/fig5_accuracy.png]

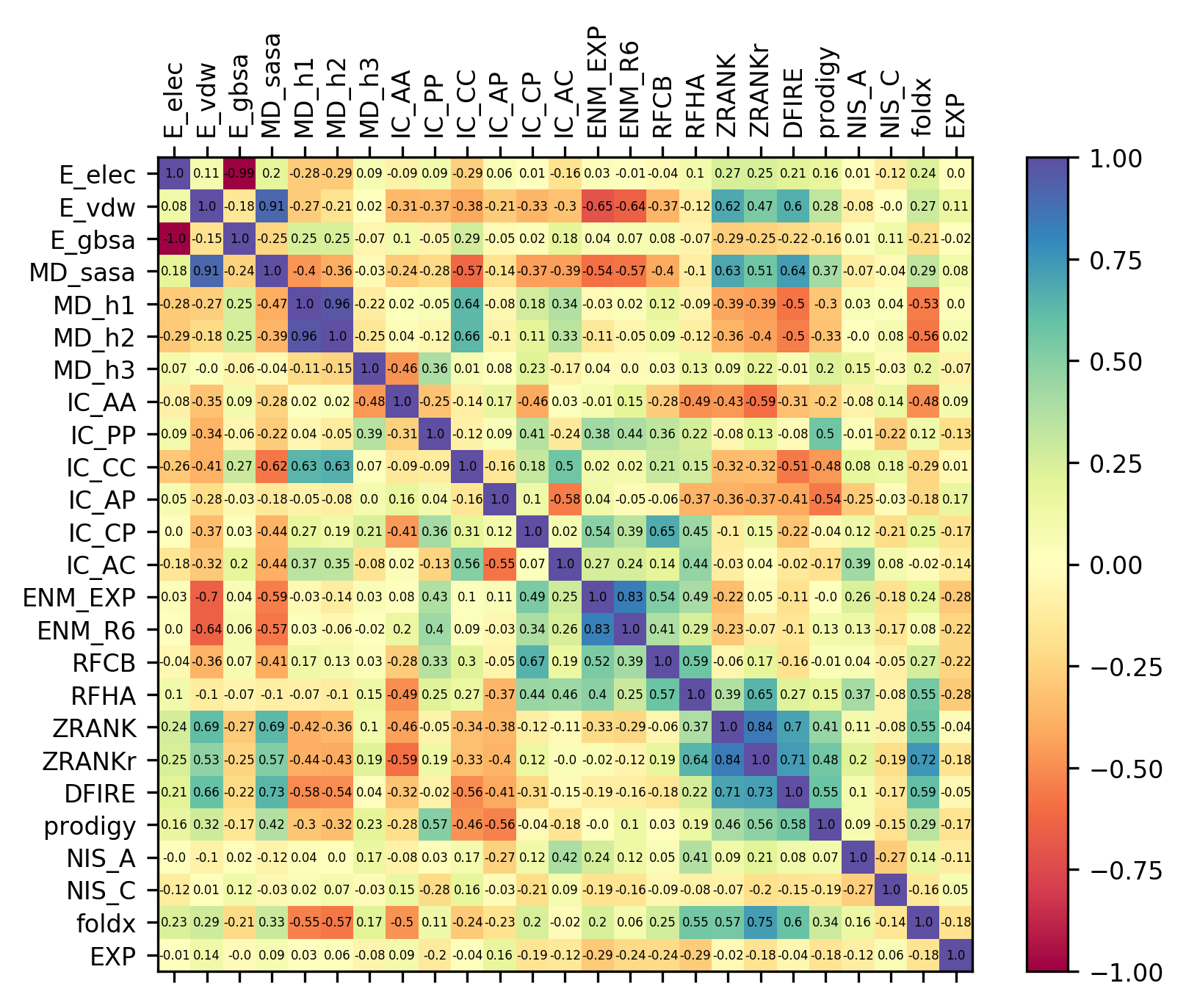

Supplement: S1 Data — (TGZ) [file pcbi.1006954.s002.tgz › supporting_data/sample_outputs/fig3.png]

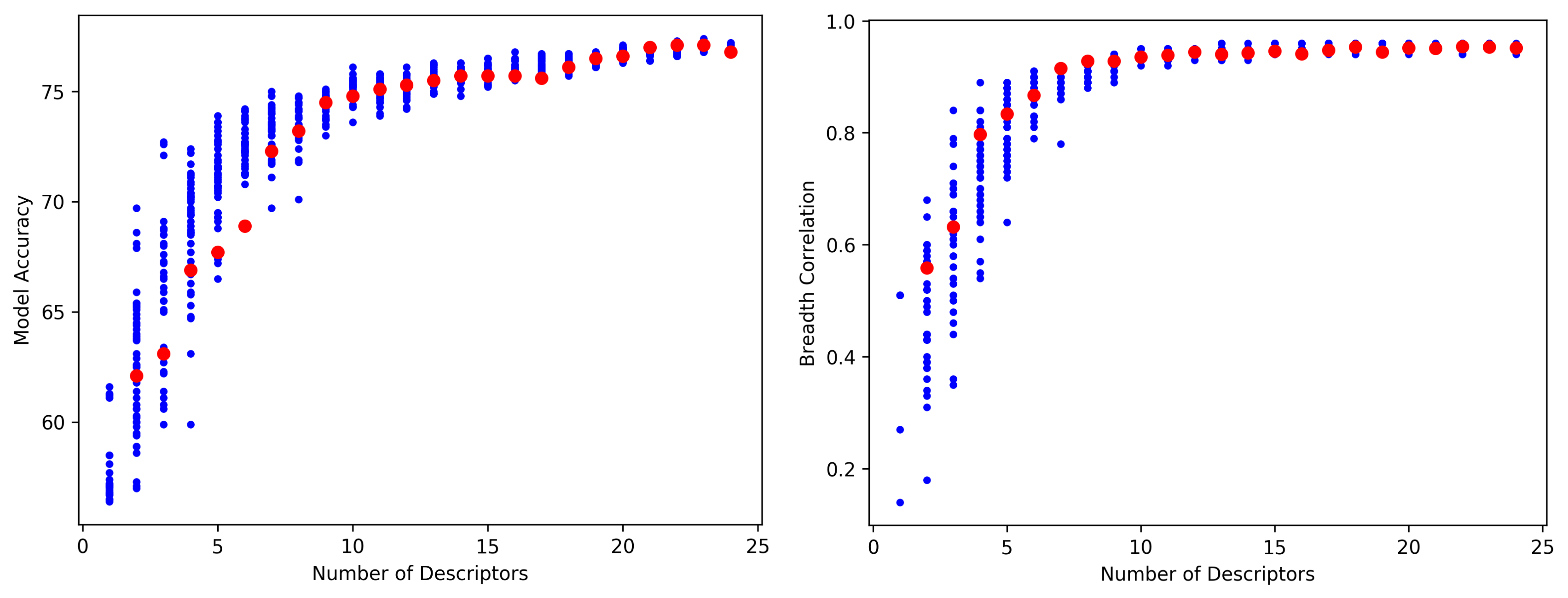

Supplement: S1 Data — (TGZ) [file pcbi.1006954.s002.tgz › supporting_data/sample_outputs/fig5.png]

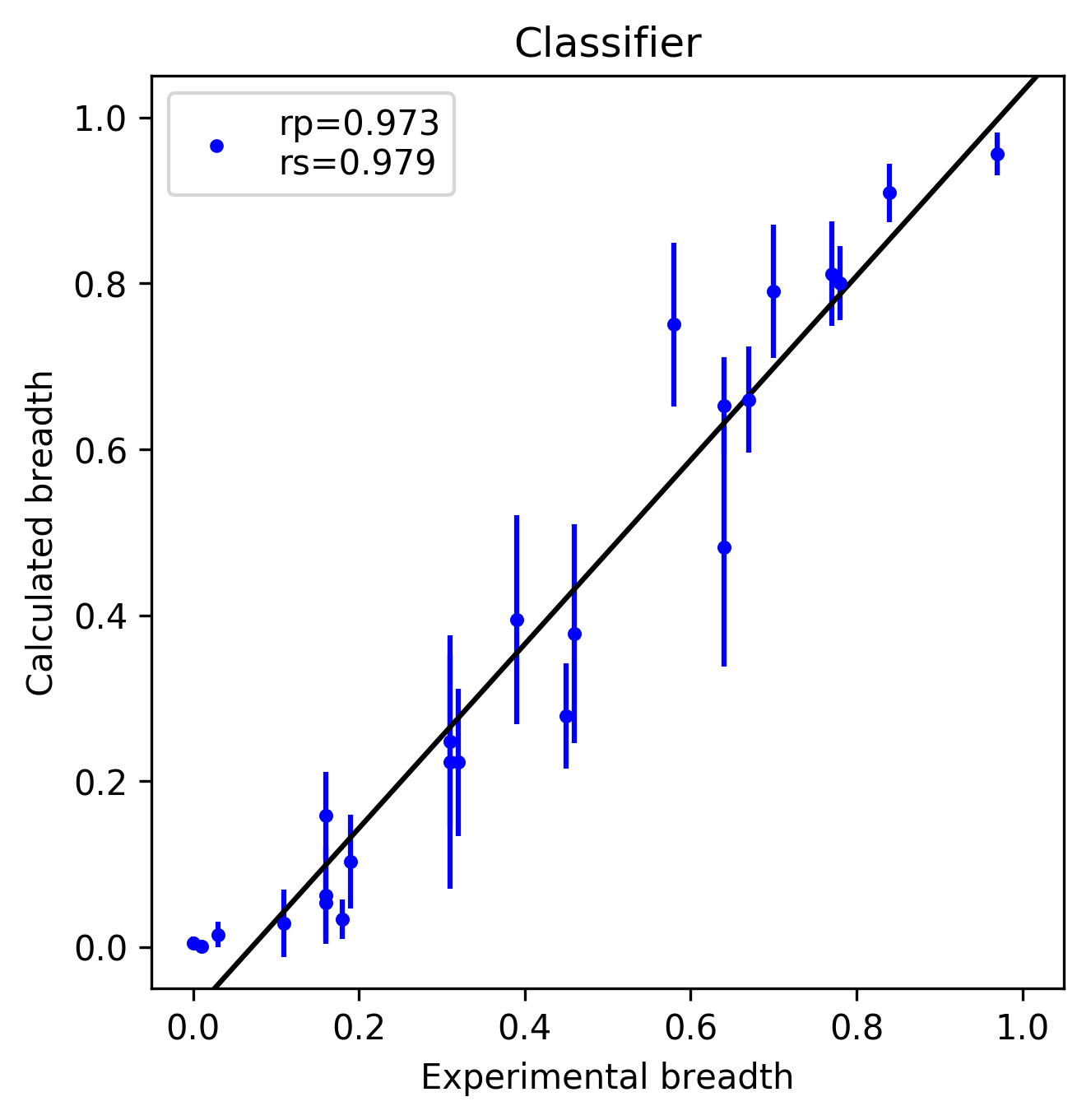

Supplement: S1 Data — (TGZ) [file pcbi.1006954.s002.tgz › supporting_data/sample_outputs/fig4_classifier.png]

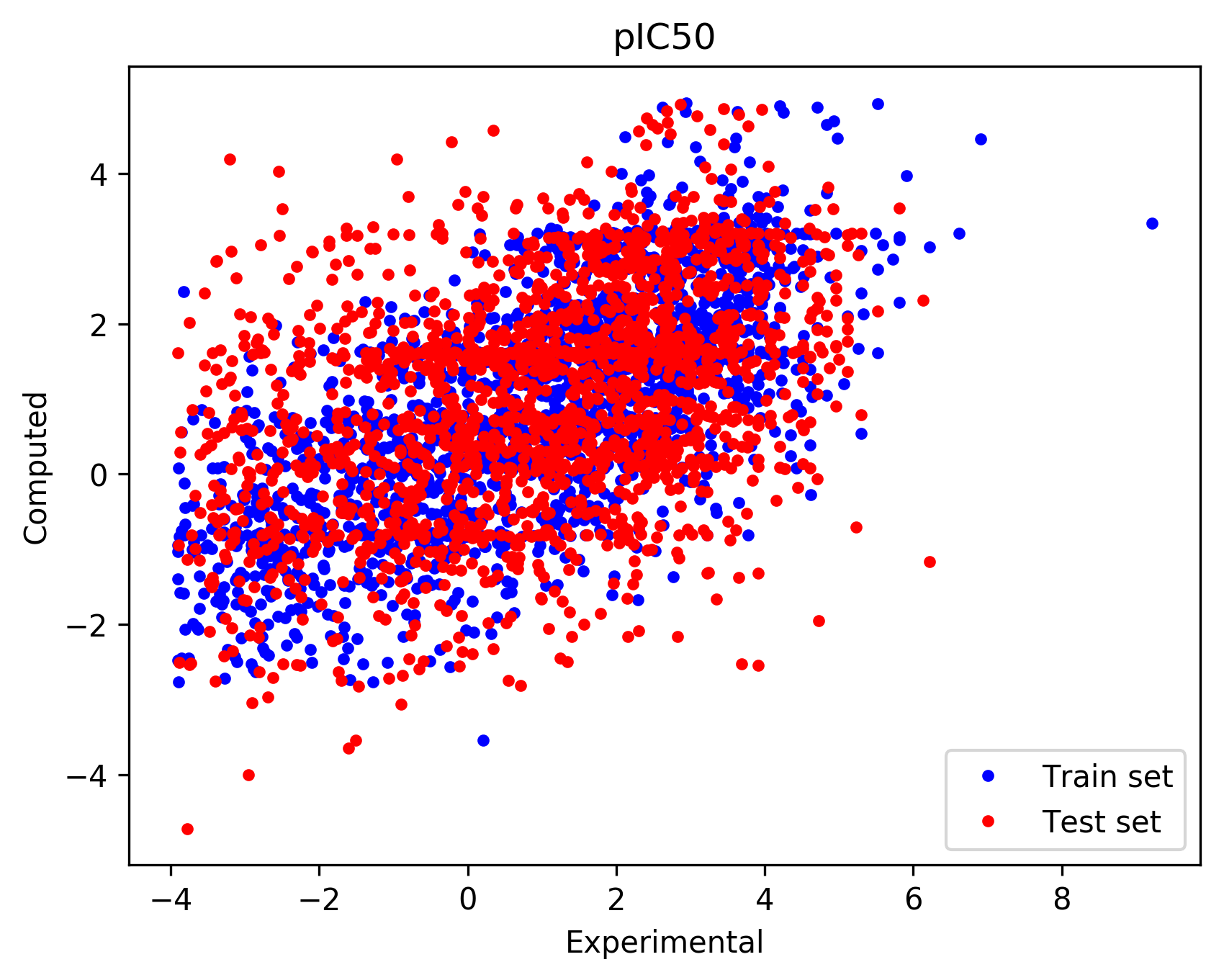

Supplement: S1 Data — (TGZ) [file pcbi.1006954.s002.tgz › supporting_data/sample_outputs/fig2.png]

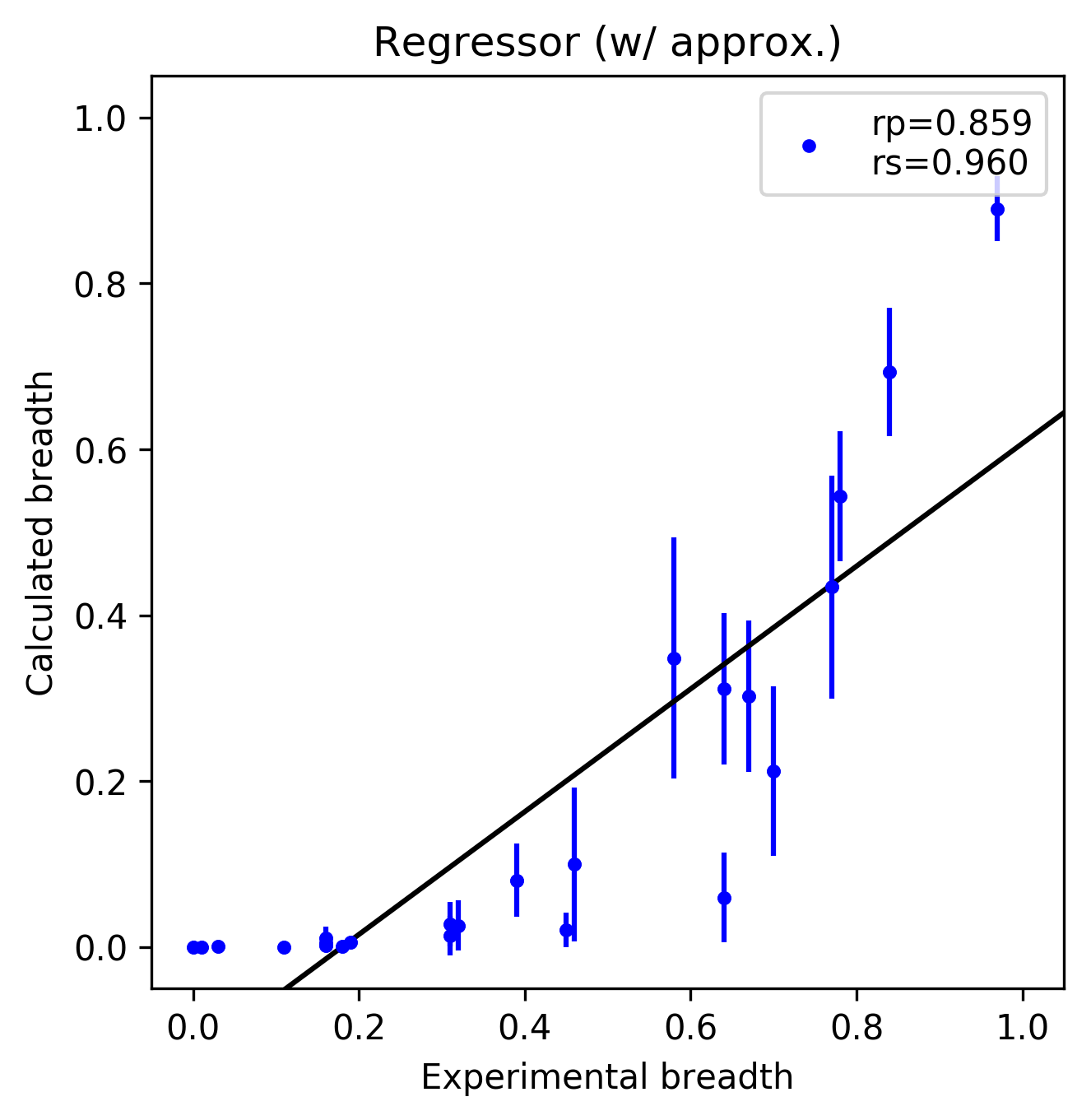

Supplement: S1 Data — (TGZ) [file pcbi.1006954.s002.tgz › supporting_data/sample_outputs/fig4_regressor_approx.png]

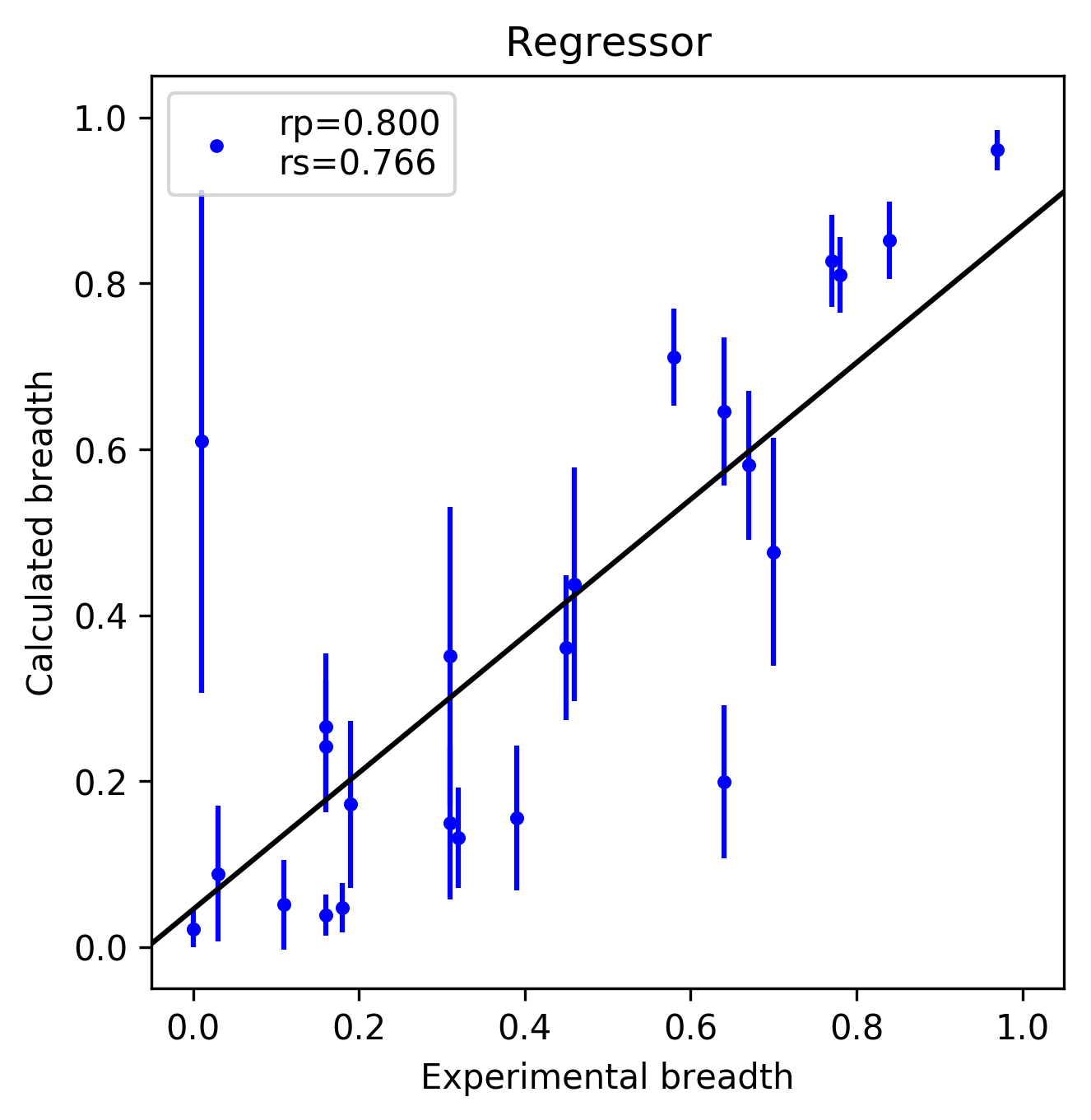

Supplement: S1 Data — (TGZ) [file pcbi.1006954.s002.tgz › supporting_data/sample_outputs/fig4_regressor.png]

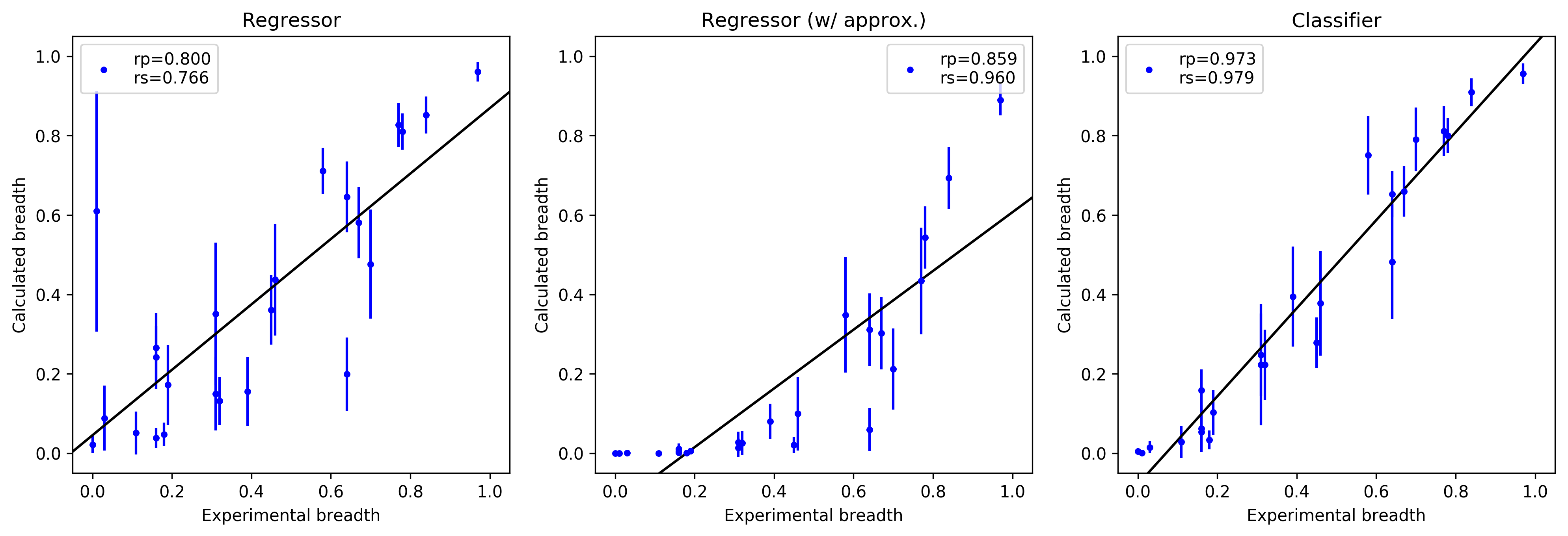

Supplement: S1 Data — (TGZ) [file pcbi.1006954.s002.tgz › supporting_data/sample_outputs/fig4.png]

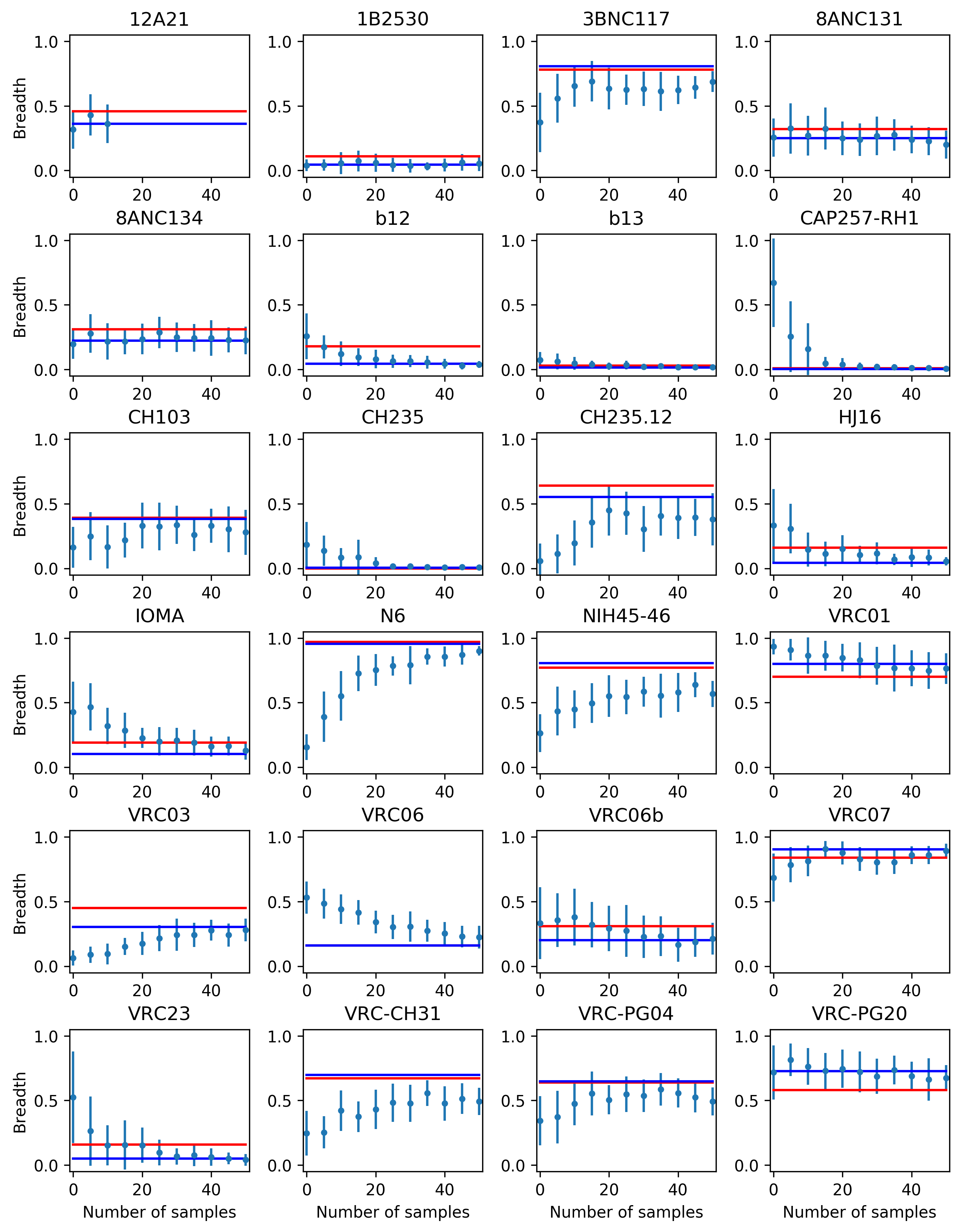

Supplement: S1 Data — (TGZ) [file pcbi.1006954.s002.tgz › supporting_data/sample_outputs/fig6.png]
